# Heavy Metals and Associated Risks of Wild Edible Mushrooms Consumption: Transfer Factor, Carcinogenic Risk, and Health Risk Index

**DOI:** 10.3390/jof10120844

**Published:** 2024-12-06

**Authors:** Ioan Alin Bucurica, Ioana Daniela Dulama, Cristiana Radulescu, Andreea Laura Banica, Sorina Geanina Stanescu

**Affiliations:** 1 Institute of Multidisciplinary Research for Science and Technology, Valahia University of Targoviste, 13 Sinaia Alley, 130004 Targoviste, Romania; bucurica_alin@icstm.ro (I.A.B.); banica.andreea@icstm.ro (A.L.B.);; 2 Faculty of Sciences and Arts, Valahia University of Targoviste, 13 Sinaia Alley, 130004 Targoviste, Romania; 3 Doctoral School Chemical Engineering and Biotechnology, National University of Science and Technology Politehnica of Bucharest, 313 Splaiul Independenței, 060042 Bucharest, Romania; 4 Academy of Romanian Scientists, 3 Ilfov Street, 030167 Bucharest, Romania

**Keywords:** wild edible mushrooms, industrial area, touristic area, Bucegi National Reservation, transfer factor, estimated daily intake, carcinogenic risk, daily intake metals, health risk index

## Abstract

This research aims to investigate the heavy metals (i.e., Cd, Cr, Cu, Ni, and Pb) in the fruiting bodies of six indigenous wild edible mushrooms including *Agaricus bisporus*, *Agaricus campestris*, *Armillaria mellea*, *Boletus edulis*, *Macrolepiota excoriate*, and *Macrolepiota procera*, correlated with various factors, such as the growth substrate, the sampling site, the species and the morphological part (i.e., cap and stipe), and their possible toxicological implications. Heavy metal concentrations in mushroom (228 samples) and soil (114 samples) were determined by Inductively Coupled Plasma—Mass Spectrometry (ICP-MS). In the first part of the study, the soil contamination (index of geo-accumulation, contamination factor, and pollution loading index) and associated risks (chronic daily dose for three exposure pathways—ingestion, dermal, and inhalation; hazard quotient of non-cancer risks and the carcinogenic risks) were calculated, while the phytoremediation capacity of the mushrooms was determined. At the end of these investigations, it was concluded that *M. procera* accumulates more Cd and Cr (32.528% and 57.906%, respectively), *M. excoriata* accumulates Cu (24.802%), *B. edulis* accumulates Ni (22.694%), and *A. mellea* accumulates Pb (18.574%), in relation to the underlying soils. There were statistically significant differences between the stipe and cap (i.e., in the cap subsamples of *M. procera*, the accumulation factor for Cd was five times higher than in the stipe subsamples). The daily intake of toxic metals related to the consumption of these mushrooms with negative consequences on human health, especially for children (1.5 times higher than for adults), was determined as well.

## 1. Introduction

Throughout time, mushrooms have been considered a valuable food source in terms of nutritional and medicinal properties [1,2,3,4,5]. On the other hand, mushrooms, which contain a high amount of organic and inorganic compounds such as carbohydrates, proteins, vitamins, and minerals have been highly recommended for therapeutic purposes [1,2,6,7] and for the treatment of various diseases, e.g., cancer [8,9,10], degenerative diseases [11,12], cardiovascular diseases [13], stress, insomnia, and depression [14], asthma [11], diabetes [15], allergies and skin diseases [11]. Remarkably, mushrooms have been used for treating complex and pandemic diseases such as acquired immunodeficiency syndrome (AIDS) [16]. Mushrooms as fresh vegetables as well as aqueous extracts possess anti-allergic [17,18], anticholesterol [19], anti-tumor [20,21], and anti-cancer [22,23] properties. Mushrooms behave as biological response modifiers on one side by promoting the positive factors and on the other eliminating the negative factors from the human body, and thus are considered as the fourth main form of conventional cancer treatment [22]. Due to the special chemical composition, including polysaccharides (e.g., β–D-glucans), vitamins, and proteins, mushrooms possess unique health-enhancing properties. In addition, fresh mushrooms are known to contain both soluble and insoluble fibers. The soluble fibers (beta-glucans polysaccharides and chitosan) are components of the cell walls and are important in preventing cardiovascular diseases. On the other hand, mushrooms can accumulate from substrate toxic elements, due to environmental pollution, including hazardous metals such as Cd, Ni, Cr, Pb, Cu, Mn, V, Zn, Hg, Sb, toxic metalloids (As) or nonmetals (Se), as well as natural radionuclide (^40^K) and anthropogenic radionuclides (^137^Cs, ^134^Cs, ^90^Sr) [24,25,26,27].

The property of mushrooms to accumulate essential elements or even toxic metals from the substrate is expressed by the bioconcentration factor (BCF), defined as the ratio of the element content in the fruiting body to the content in an underlying substrate. A series of studies [28,29,30,31,32,33], including previous research of authors [25,26,34,35,36,37], reported that wild edible or poisonous mushrooms accumulate extremely high concentrations of metals in both cap and stipe, respectively. Radulescu et al. [25,34,35,38] reported the highest content of toxic metals (i.e., Cd, Ni, Cr, Pb, Cu, Mn) in the fruiting body of different species of mushrooms (over 30 wild edible species, e.g., *Armillaria* sp., *Boletus* sp., *Russula* sp., *Amanita* sp., *Fistulina* sp., *Cantharellus* sp., *Agaricus* sp., *Lycoperdon* sp., *Macrolepiota* sp., *Pleurotus* sp., *Lactarius* sp., *Russula* sp., *Tricholoma* sp., *Lentinus tigrinus*) taking into account the following factors: various steps of growing (youthfulness 1–7 days; maturity between 20 days), two types of habitat (soil and tree bark), geographical position (hill or mountain forest, plain), type of pollution (industrial activities or forest on the side of the road with high traffic and natural park), type of people (who frequently consume or who trade), location (rural or urban), and anatomical features (size, color, type of pileus, etc.). Studies have been surprising, whereby each studied mushroom species revealed at least one unique characteristic regarding chemical composition, behavior towards metal (different for cap compared with stipe), and bioaccumulation factor. Studies reported that typical elements accumulated in mushrooms (BAF > 1) include Au, Ag, As, Br, Cd, Cs, Cu, Hg, Rb, Se, V, Zn, and Cl. Elements with typically low concentrations in macrofungi (BAF < 1) include Co, Cr, F, I, Ni, Sb, Sn, Th, U, and rare earth elements [33]. The health risk index was widely used as a promising tool to assess food [39,40,41,42,43,44] and water [39,45,46,47] contamination.

In some studies, it was reported that macrofungi (i.e., mushrooms) are rich in nutrients and beneficial health components, but they have a good capacity to accumulate heavy metals—making them both a potential health risk and a useful tool for bioremediation [48,49,50,51,52]. Liu et al. sustain that it is not clear the enrichment characteristics and functions of heavy metal transporters in the accumulation of HMs in edible fungi [53]. In the case of cultivated mushrooms, the main human health issues related to mushroom consumption are due to the excessive usage of chemical pesticide, mineral fertilizer, fluorescent whitening agents, sulfur dioxide, food additives, or HMs-polluted environment (i.e., contaminated substrate: processing residues of sawdust, cotton seed shell, corn cob, straw, bran, and other green plants) [54]. In the case of wild edible mushrooms, the HMs accumulation is species-specific (i.e., it is correlated with the species’ physiology) and depends on the environment of the collection site, i.e., the soil’s mineral content and the distance from the pollution sources [52,55]. In the complex study of Falandysz et al., the effect of the developmental stage on allocation and sequestration of metallic and metalloid elements in cap and stipe was determined in the case of *Amanita muscaria* (L.) mushrooms [56]. They established six developmental stages of the studied mushroom and analyzed the mineral nutrients and environmental pollutants in order to determine if these elements affect the fungi growth [56]. As a remark, the metabolic and nutritional state of the fungi organism determines all mechanisms related to absorption, translocation, and accumulation of metallic and metalloid elements in macromycetes’ fruiting bodies [56].

Regarding originality and novelty, this study is the first one that evaluates the heavy metals accumulation (i.e., Cd, Cr, Cu, Ni, and Pb) in soils and fruiting bodies of the most consumed indigenous wild edible mushrooms in Prahova County, correlated with the studied area. At the same time, it was the first time that the area—industrial and touristic cities—was analyzed from the point of view of pollution impact on wild mushrooms, considering that three of the studied cities are at the limit of Bucegi Natural Park. People harvest these mushrooms for consumption, believing that if they grew in the natural park area, it is in a clean, pollution-free area. This study provides valuable information for authorities to develop and implement some strategies and legislation for promoting public health and reducing environmental pollution in tourist areas. Furthermore, for the industrial areas or transition zones, citizens should be informed about the risk of consuming wild mushrooms grown on historically contaminated soils.

## 2. Materials and Methods

### 2.1. Sampling and Sample

**Figure 1:**
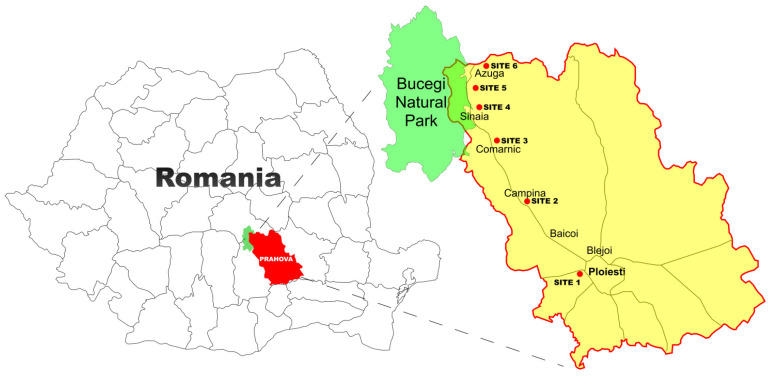
Sampling sites.

The wild edible indigenous species (i.e., *Agaricus bisporus*, *Agaricus campestris*, *Armillaria mellea*, *Boletus edulis*, *Macrolepiota excoriata*, and *Macrolepiota procera*) used in this study were collected from Prahova County in the summer and autumn of 2023 (Figure 1). The mushroom species chosen for this study were characterized from morphological point of view and nutritional value in a previous study [26]. The sampling sites (two industrial cities, three touristic cities—at the limit with the Bucegi National Reservation—and another one in the transition area) were chosen in order to evaluate the health risk of mushroom consumption depending on their region and species.

The analyzed samples in this study were chosen because they are among the most consumed species, the species grow both in the plains (Sites 1 and 2), but also in the mountainous and pre-mountainous areas (Sites 3–6), and they are easy to identify and distinguish from the poisonous ones. The mushroom samples were collected manually using non-powdered protective gloves and cleaned with a soft brush (made of natural fibers) to remove the vegetal and soil wastes. After that, the mushrooms were separated by cap and stipe subsamples and were packaged in coded sterile polyethylene vials to be transferred to the laboratory. The soil samples (substrate of the mushroom samples) were collected according to the instructions presented in LUCAS 2009/2012 [57], from the surface to a maximum of 10 cm depth using plastic shovels and stored in coded sterile plastic bags. All data about the collected samples are presented in Table 1.

### 2.2. Reagents

All chemical reagents used in this study were of analytical grade. Moreover, distilled deionized water (Milli-Q Water System Millipore, Burlington, VT, USA) was used throughout. Nitric acid (65%, Merck, Rahway, NJ, USA), hydrochloric acid (37%, Merck), and hydrogen peroxide (30%, Merck) were used for the microwave digestion process and blanks preparation as well. For calibration curves, the ICP multi-element standard solution IV certified reference material (23 elements in HNO_3_ Suprapur^®^ 6.5%, Merck) was used.

### 2.3. Sample Preparation

The collected fresh mushrooms were carefully cleaned of wastes (i.e., soil and vegetal) with deionized water and were cut in small pieces with a plastic knife. All samples (mushroom subsamples and soil) were dried at 40 °C for 48 h, until they reached the constant weight. After that, the samples free of moisture were grinded in order to obtain a fine and homogenous powder. These samples, as well as the blank samples, were subjected to the microwave digestion process presented in Table 2.

### 2.4. Inductively Coupled Plasma—Mass Spectrometry (ICP-MS)

The clear solution samples, obtained as it was described in the previous section, were analyzed by Inductively Coupled Plasma—Mass Spectrometry (ICP-MS) to determine the heavy metals content (i.e., Cd, Cr, Cu, Ni, and Pb), For this purpose, the iCAP™ Qc mass-spectrometer (Thermo Scientific, Darmstadt, Germany) was used. The measurements were achieved in triplicate in the standard mode (STD), using the Qtegra Intelligent Scientific Data Solution. The relative standard deviation (RSD) values were less than 10%; the data were expressed as mg·kg^−1^ dried weight (d.w.) material. The quantification of this technique was performed by a standard curve procedure using certified reference material. Metals calibration curves showed good linearity over the concentration range (0.1 to 10.0 mg·L^−1^), with R^2^ correlation coefficients in the range of 0.997 to 0.999. The limits of detection (LODs) and limits of quantitation (LOQs) of analyzed elements were established using the calibration data, and standard reference materials (i.e., NIST SRM 1515 Apple leaves and SRM 2710a Montana I Soil) were used to verify the accuracy and traceability of the method (Table 3).

### 2.5. Data Analysis of Soil Samples

#### 2.5.1. Index of Geo-Accumulation

The ICP-MS data represent the first sequence in establishing the contamination level of the studied soils. In this respect, the index of geo-accumulation (*I_geo_*) was calculated according to Equation (1) proposed by Muller [58] and reported by Birch [59]:

(1) $$I_{geo}={log}_{2}\frac{C_{n}}{1.5\cdot B_{n}}$$

where *C_n_* = the recorded metal content, 1.5 = the correction factor (due to variation in background values and lithogenic effects) [60], and *B_n_* = the background values for sedimentary rocks (Cd = 0.035 mg·kg^−1^; Cr = 11 mg·kg^−1^; Cu = 4 mg·kg^−1^; Ni = 20 mg·kg^−1^; Pb = 9 mg·kg^−1^) [61]. The obtained data are presented in Table 2. According to the Muller scale, the value of I_geo_ establishes the pollution class of the soil sample: class 0, I_geo_ < 0 unpolluted; class 1, 0 < I_geo_ < 1 unpolluted to moderately polluted; class 2, 1 < I_geo_ < 2 moderately polluted; class 3, 2 < I_geo_ < 3 moderately to strongly polluted; class 4, 3 < I_geo_ < 4 strongly polluted; class 5, 4 < I_geo_ < 5 strongly polluted; class 6, 5 < I_geo_ extremely polluted [62].

#### 2.5.2. Contamination Factor (CF) and Pollution Loading Index (PLI)

The contamination factor (CF) represents a singular index calculated as a ratio between the element concentration in the soil and the normal values in the study area—Equation (2). In this case, the normal values are regulated by the Romanian Order no. 756/1997 [63]. The pollution loading index was used to determine the overall level of heavy metals toxicity in a soil sample and was calculated using Equation (3):

(2) $${CF}_{x}=\frac{C_{x}}{NV}$$

where *CF_x_* = the contamination factor of the metal *x*; *C_x_* = the value of the metal *x* concentration; and *NV* = the normal values in the studied area. The *CF* classes are as follows: low contamination (*CF* < 1), moderate contamination (1 ≤ *CF* < 3), considerable contamination (3 ≤ *CF* < 6), and very high contamination (*CF* ≥ 6) [64].

(3) $$PLI={\left({CF}_{1}\cdot {CF}_{2}\cdot \ldots \cdot {CF}_{n}\right)}^{1/n}$$

where *PLI* = the pollution loading index; *CF_x_* = the contamination factor of the metal *x* (calculated according to Equation (2)); *n* = the number of the determined metals. The evaluation of the soil pollution level using *PLI*: 0 < *PLI* ≤ 1 unpolluted; 1 < *PLI* ≤ 2 moderately to unpolluted; 2 < *PLI* ≤ 3 moderately polluted; 3 < *PLI* ≤ 4 moderately to highly polluted; 4 < *PLI* ≤ 5 highly polluted; *PLI* > 5 very highly polluted [65].

#### 2.5.3. Exposure Assessment and Health Risk Evaluation

The health risk associated with using contaminated soil by adults and children was assessed by the following:

- Ingestion exposure:

(4) $$D_{ing}=\frac{C\cdot IR\cdot ED\cdot EF}{BW\cdot AT}\cdot CF$$

where *D_ing_* = the chronic daily dose via ingestion (expressed as mg·kg^−1^·day^−1^); *C* = concentration of the metal (expressed as mg·kg^−1^); *IR* = ingestion rate: for adults 100 mg·day^−1^, for children 200 mg·day^−1^; *ED* = exposure duration: for adults 20 years, for children 6 years; *EF* = exposure frequency: for adults and children 365 days·year^−1^; *BW* = body weight: for adults 70 kg, for children 15 kg; *AT* = average time: for adults 7300 days, for children 2190 days; and *CF* = conversion factor: for adults and children 1 × 10^–6^ [66,67].

- Inhalation exposure:

(5) $$D_{inh}=\frac{C\cdot InhR\cdot ED\cdot EF}{BW\cdot AT\cdot PEF}$$

where *D_inh_* = the chronic daily dose via inhalation (expressed as mg·kg^−1^·day^−1^); *C* = concentration of the metal (expressed as mg·kg^−1^); *InhR* = inhalation rate: for adults 20 m^3^·day^−1^, for children 7.6 m^3^·day^−1^; *ED* = exposure duration: for adults 20 years, for children 6 years; *EF* = exposure frequency: for adults and children 365 days·year^−1^; *BW* = body weight: for adults 70 kg, for children 15 kg; *AT* = average time: for adults 7300 days, for children 2190 days; and *PEF* = particle emission factor: for adults and children 1.36 × 10^9^ m^3^·kg^−1^ [66,67].

- Dermal exposure:

(6) $$D_{dermal}=\frac{C\cdot SA\cdot SAF\cdot DAF\cdot ED\cdot EF}{BW\cdot AT}\cdot CF$$

where *D_dermal_* = the chronic daily dose via dermal exposure (expressed as mg·kg^−1^·day^−1^); *C* = concentration of the metal (expressed as mg·kg^−1^); SA = surface area: for adults 6032 cm^2^, for children 2373 cm^2^; SAF = Specific Adjustment Factor; DAF = Dosimetric Adjustment Factor; *ED* = exposure duration: for adults 20 years, for children 6 years; *EF* = exposure frequency: for adults and children 365 days·year^−1^; *BW* = body weight: for adults 70 kg, for children 15 kg; *AT* = average time: for adults 7300 days, for children 2190 days; and *CF* = conversion factor: for adults and children 1 × 10^–6^ [66,67].

The total exposure (*D_total_*, expresses as mg·kg^−1^·day^−1^) was calculated as the sum of ingestion, inhalation, and dermal exposure:

(7) $$D_{total}=D_{ing}+D_{inh}+D_{dermal}$$

The hazard quotient for non-cancer risks was estimated using Equations (8)–(10):

(8) $${HQ}_{ing}=\frac{D_{ing}}{{RfD}_{ing}}$$

(9) $${HQ}_{inh}=\frac{D_{inh}}{{RfD}_{inh}}$$

(10) $${HQ}_{dermal}=\frac{D_{ing}}{{RfD}_{dermal}}$$

(11) $${HI}_{nCR}={HQ}_{ing}+{HQ}_{inh}+{HQ}_{dermal}$$

where *RfD* = reference dose (values are presented in Table 4).

**Table 4:** Reference dose (*RfD*) values used for hazard quotient determination [66].

*RfD*	Cd	Cr	Cu	Ni	Pb
Ingestion	0.001	0.003	0.04	0.02	0.0014
Inhalation	0.0004	0.0000286	0.0402	0.0206	0.00325
Dermal	0.000025	0.003	0.012	0.00054	0.000524

The cancer risk was estimated using Equations (12)–(14):

(12) $${CR}_{ing}=D_{ing}\cdot {CSF}_{ing}$$

(13) $${CR}_{inh}=D_{inh}\cdot {CSF}_{inh}$$

(14) $${CR}_{dermal}=D_{dermal}\cdot {CSF}_{dermal}$$

(15) $${HI}_{CR}={CR}_{ing}+{CR}_{inh}+{CR}_{dermal}$$

where *CSF* = cancer slope factor (values are presented in Table 5).

**Table 5:** Cancer slope factor (*CSF*) values used for cancer risk determination [66].

*CSF*	Cd	Cr	Ni	Pb
Ingestion	6.30	0.5	0.91	0.0085
Inhalation	6.01	4.1	0.84	0.0420
Dermal	-	2.0	4.25	-

CSF

Cd

Cr

Ni

Pb

### 2.6. Data Analysis of Mushroom Samples

#### 2.6.1. Phytoremediation Capacity—Transfer Factor (TF)

Transfer factor (TF) represents the capacity of the plant (in this case, the capacity of mushrooms) to transfer the metals from soil in their anatomic parts. It is easily calculated as the ratio between metal content in mushroom parts (i.e., cap and stipe) and metal content in soil [27,40,68], expressed in [%]. This factor can explain the risks of human exposure to contaminated soils [41] and the capacity of some species to hyper-accumulate metals, and so, it can be determined if they can be used in soil bioremediation [42].

#### 2.6.2. Health Risk Assessment

Estimated daily intake (*EDI*) represents the metals ingested daily (expressed as mg·day^−1^) into the human body of a consumer, without taking into account the ejected metals due to the metabolic processes [40]. This indicator is calculated using the following formula:

(16) $$EDI=C_{mushroom}\times I$$

where *C_mushroom_* represents the metal content in mushroom (mg·kg^−1^ d.w.) and *I* represent the daily intake rate of the mushrooms (i.e., 0.1 kg·day^−1^ d.w. for adults and 0.03 kg·day^−1^ d.w. for children).

Carcinogenic risk associated with lead exposure (*CR_Pb_*) represents the estimation of the probability of developing a cancer form after exposure to Pb sources. The *CR* can also be estimated for As exposure [43], but this element was not determined for this study. In this respect, *CR_Pb_* was calculated using the following formula:

(17) $${CR}_{Pb}=EDI\times CSF$$

where *CSF* represents the cancer slope factor (i.e., 0.0085 mg^−1^·kg·day). The US EPA establishes the acceptable risk levels for *CR_Pb_* between 10^−6^ and 10^−4^ [44].

Daily intake metals (*DIM*), expressed in mg·kg^−1^·day^−1^, was calculated using following formula:

(18) $$DIM=\frac{EDI}{BM}$$

where *BM* represents the average body mass of a standardized person (i.e., 70 kg for adults and 14 kg for children).

Health risk index (HRI) was used to estimate the health risks induced by the consumption of contaminated mushrooms. This indicator takes into account the *DIM* (calculated according to the previous formula) and the oral reference dose established by the US EPA: 0.001 mg·kg^−1^·day^−1^ for Cd, 0.003 mg·kg^−1^·day^−1^ for Cr(VI), 1 mg·kg^−1^·day^−1^ for Cr(III), 0.040 mg·kg^−1^·day^−1^ for Cu, 0.020 mg·kg^−1^·day^−1^ for Ni, and 0.035 mg·kg^−1^·day^−1^ for Pb [40,43,44,45,46,47,69].

### 2.7. Statistical Processing of the Obtained Data

For the basic statistical processing of the obtained data (i.e., minimum, maximum, average value etc.), the functions of Microsoft Office Excel were used. For an advance statistical analysis of the data, IBM SPSS Statistics software (v.26) was used to interpret the descriptive data, to perform correlation tests, and to use advanced data modeling methods through cluster analysis.

## 3. Results and Discussion

Eight heavy metals (i.e., Cu, Cr, Cd, Pb, Zn, Ni, Hg, and As) are considered by the United States Environmental Protection Agency (US EPA) as the most common harmful heavy metals in the environment [69]. Heavy metals (HMs) can be found in the environment in different forms: vapors, dissolved ions in water, and salts/minerals (in rock, soil, dust, and sand), among other forms. Moreover, they can be found in both organic and inorganic molecules and may adhere to airborne particles [29]. Therefore, heavy metals coming from many sources are accumulated in mushrooms [25,26,29,61,70,71,72], and because HMs are so persistent in the environment, there may be a significant concern for human health.

### 3.1. Heavy Metals in Soil Samples

The mean values of the HM content in soil samples are shown in Table 6 (full data are presented in Supplementary Material S1—Soil samples). The concentrations of Cd, Cr, Cu, Ni, and Pb ranged from 2.324 to 6.416 mg·kg^−1^, 1.360 to 5.773 mg·kg^−1^, 11.804 to 43.970 mg·kg^−1^, 5.099 to 13.464 mg·kg^−1^, and 8.355 to 18.102 mg·kg^−1^, respectively, with mean contents of 4.359 mg·kg^−1^, 3.462 mg·kg^−1^, 16.041 mg·kg^−1^, 8.991 mg·kg^−1^, and 10.432 mg·kg^−1^, respectively. It can be easily highlighted that the content of Cd was higher than the normal level established by the Romanian Ministry of Waters, Forests and Environmental Protection (Order 756/1997, updated in 2011) [63]; thus, this element requires intensive monitoring to prevent further accumulation. The fact that Cd and Cu have been recorded in concentrations higher than the European average value in topsoils [61] represents another worrying aspect.

**Table 6:** Mean values of elements (i.e., Cd, Cr, Cu, Ni, and Pb) determined in soil samples in terms of both species and collecting sites (mg·kg^−1^ d.w.), compared with values recommended by Romanian Order no. 756/1997 [63] and European average value [61].

	Cd	Cr	Cu	Ni	Pb
Collecting sites					
Site 1 (n = 24)	3.800	3.824	21.248	11.257	12.532
Site 2 (n = 24)	4.386	3.667	13.174	9.264	9.926
Site 3 (n = 18)	4.984	2.531	20.713	6.841	9.740
Site 4 (n = 18)	4.156	4.127	12.429	7.733	9.926
Site 5 (n = 18)	4.149	2.871	13.289	10.026	10.533
Site 6 (n = 12)	5.104	3.615	13.903	7.476	8.890
Mushroom species					
*Agaricus bisporus* (n = 24)	4.766	3.478	17.625	8.181	9.273
*Agaricus campestris* (n = 12)	4.729	3.910	13.202	11.033	9.212
*Armillaria mellea* (n = 36)	5.261	3.176	19.912	9.509	10.959
*Boletus edulis* (n = 18)	2.999	3.528	12.379	7.144	11.354
*Macrolepiota excoriata* (n = 12)	3.454	4.825	11.841	8.033	9.587
*Macrolepiota procera* (n = 12)	3.412	2.378	13.796	10.749	11.852
Values recommended by Romanian Order no. 756/1997 [63]					
Normal values	1.000	30.000	20.000	20.000	20.000
Maximum threshold	3.000	100.000	100.000	75.000	50.000
Intervention threshold	5.000	300.000	200.000	150.000	100.000
Statistical data of analytical results for topsoil (European average value) [61]					
	0.145	22.000	13.000	37.300	22.600

The highest values for Cu, Ni, and Pb were recorded in the Ss11 sample as well as the mean values of all soil samples collected in Ploiesti City (Site 1). This is the result of the historical pollution, due to the intensive industrial (oil industry—refineries; food, beverage, and tobacco industry; building and building materials industry; etc.) and agricultural activities over time. Site 3 (Comarnic City) was in the category of industrial cities until 25 years ago, with the main polluters being cement and brick factories, as well as the furniture factory. At present, because the main road (National Road 1/ European Road E60) passes through the middle of the city, the car traffic represents the main pollution source. The highest level of cadmium recorded in Site 6 (Azuga City) can be explained by the former industrial activities carried out in the glass, textile, lime, cement, and timber factories, while the highest value for chromium was recorded in Site 4 (Sinaia City). It must be mentioned that in this site, the fine mechanics factory still exists, which includes activities such as chrome, nickel, and zinc plating, as well as metal prototyping.

### 3.2. Ecological Indices

#### 3.2.1. Index of Geo-Accumulation

**Figure 2:**
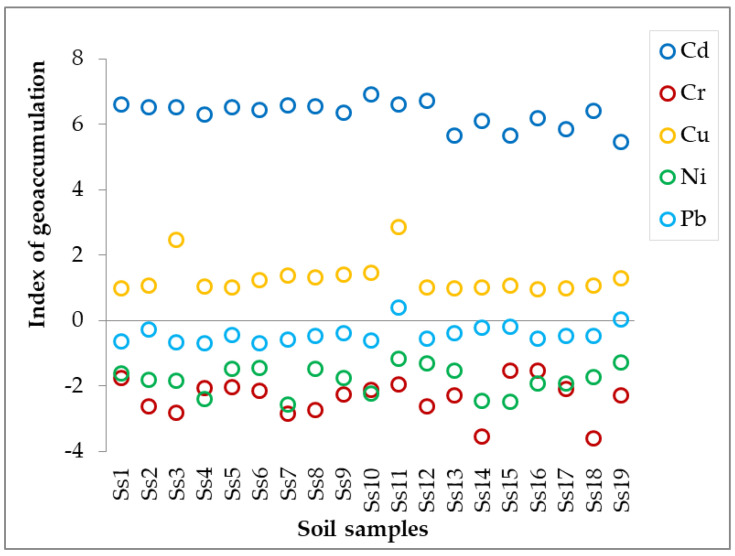
Index of geo-accumulation calculated for the analyzed soil samples.

Figure 2 (and Supplementary Material S1—Soil samples) presents the geo-accumulation index (*I_geo_*) of HMs within the studied area. For cadmium, the *I_geo_* values exceeded value 6 in 16 samples and 5 in 3 samples, indicating extreme pollution of this metal, while for copper, *I_geo_* values exceeded value 2 in 2 samples and value 1 in 14 samples (the values for the remaining 3 samples were close to value 1), indicating moderate pollution. In the case of chromium, nickel, and lead, the *I_geo_* values were negative—this suggests a low-level contamination.

Taking into account the soil distribution in terms of collecting sites (Table 7 and Supplementary Material S1—Soil samples), it can be easily seen that all sites are extremely polluted with cadmium and moderately polluted with copper. The same observation can be made for the soil distribution in terms of mushroom species: all soils are extremely polluted with cadmium and moderately polluted with copper (except the *M. excoriata* which recorded a mean value of 0.981).

The average *I_geo_* revealed that all the examined samples fell into class 6, indicating extremely contaminated soils because of the high values of *I_geo_* of Cu, Ni, and Pb. For the other analyzed metals, the *I_geo_* values are in the range of −3.026 to −3.837 for Cd, which highlights uncontaminated soils, and 4.124 to 5.145 for Cr, which highlights strong to extreme contamination. These high values are caused mainly by industry; at present, Sites 1 and 2 still have industrial activity, but the other sites (i.e., 3–6) are recognized as tourist areas where car traffic and anthropic activity are the main polluting sources. The index of geo-accumulation in the areas affected by industrial activity may be elevated in the next years due to the expansion of industries and the growing number of cars.

#### 3.2.2. Contamination Factor (CF) and Pollution Loading Index (PLI)

**Figure 3:**
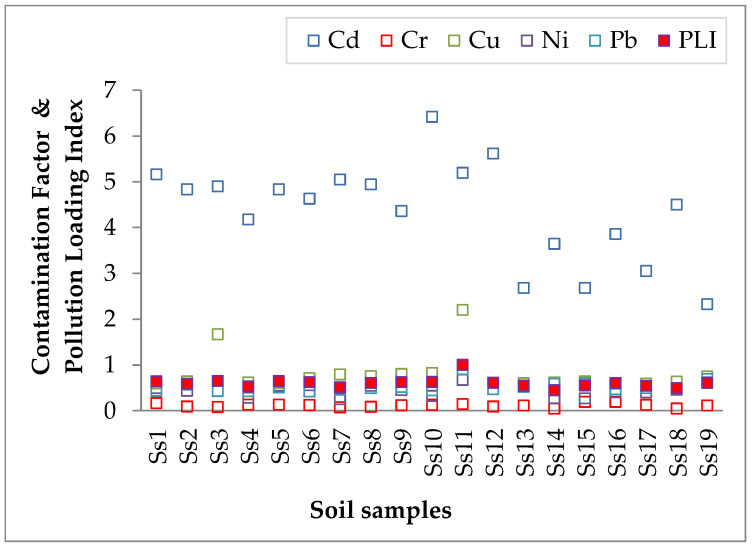
Contamination factor (*CF*—empty squares) and pollution loading index (*PLI*—red full square) of the heavy metals within the studied area.

The obtained results for the contamination factors of the soil samples are shown in Figure 3 and Supplementary Material S1—Soil samples. The *CF* of Cr, Cu, Ni, and Pb were within low contamination (1 > *CF*) for the entire studied area (except Ss3 and Ss11, with *CF_Cu_* 1.667 and 2.198, respectively). The Cd *CF* values for the analyzed samples indicate considerable contamination (3 ≤ *CF* < 6) for 16 samples, while the other values indicate moderate contamination (1 ≤ *CF* < 3).

Regarding the *PLI* values of the soil samples (shown in Figure 3 and Supplementary Material S1—Soil samples), the results revealed that the *PLI* does not exceed 1, indicating that the soil is unpolluted.

#### 3.2.3. Exposure Assessment

Table 8 shows the chronic daily dose calculated for three exposure pathways (ingestion, dermal, and inhalation) and the total value for adults and children. The trend for both categories (adults and children) was found in order of ingestion > dermal > inhalation.

The inhalation exposure route presents the lowest dose among the three exposure routes determined with 3–4 orders of increase, comparatively with ingestion or dermal routes, which were the major pathways for both categories. In the case of cadmium, the total values for chronic daily dose were higher in Sites 6 and 3, while in the case of chromium, the maximum total value for chronic daily dose was recorded in Site 4. Table 3 highlights the maximum value for copper and lead in Site 1 and for nickel in Site 3. The values for adults were lower than the values for children across both sampling locations and mushroom species (Supplementary Material S1—Soil samples). These results are in accordance with other scientific research demonstrating that children are more exposed than adults [66,73].

#### 3.2.4. Health Risk Evaluation

The hazard quotient of non-cancer risks (*HQ* and *HQ_total_*) and the carcinogenic risks (*CR* and *HI_total_*) represent the main components for a complete health risk assessment. All calculated data are available in Supplementary Material S1—Soil samples, and the total values of hazard quotient of non-cancer risks and hazard index of cancer risks calculated for adults and children, in terms of both species and collecting sites, are shown in Table 9.

The *HQ* results for the studied samples were higher than the *HI* values for both categories (i.e., adults and children). Taking into account the fact that a *HI* value lower than 1 indicates a low non-cancer risk to both age categories [74,75], as expected, the values for adults were lower than the values for children across both sampling locations and mushroom species (Table 9 and Supplementary Material S1—Soil samples). These results are in agreement with previously reported data [75] and demonstrate that children are more exposed than adults.

In terms of mushroom species, it can be easily observed that *A. mellea* induces the highest total risk for human health (for both age categories and in terms of cancer and non-cancer risks) due to the high values of risks induced by cadmium, copper, and lead. The *HI* values < 10^–6^ represent a low risk, while values > 10^–4^ mean a high risk to human health [76]. As a general observation, the *HI* results were higher than 10^–4^ for the children, indicating a likely cancer risk [77], while the *HI* values higher than 10^–5^ for adults were within a tolerable limit.

### 3.3. Heavy Metals in Mushroom Samples

The results obtained in the study of elemental content (i.e., Cd, Cr, Cu, Ni, and Pb) of 128 analyzed mushroom subsamples are shown in Table 10. Regarding the cadmium and lead contents, all the samples exceed the maximum permitted limits for vegetables [78,79,80] and seem to pose a health risk for people (adults and children). Regarding the copper content, it should be noted that all the samples comply with the maximum permitted limit established by the Romanian order [78]. Taking into account that the Commission Regulation (EC) no. 2023/915 establishes maximum levels for Cd and Pb in wild fungi (i.e., 0.500 mg·kg^−1^ and 0.800 mg·kg^−1^, respectively) [81], values which are highest comparatively with other regulations, it was highlighted that in all mushroom stipe subsamples, except *A. mellea*, values were recorded that comply with the maximum permitted limits.

### 3.4. Phytoremediation Capacity—Transfer Factor (TF)

**Figure 4:**
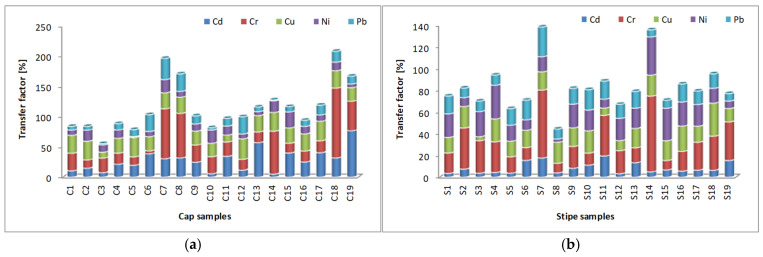
The transfer factor in cap (**a**) and stipe subsamples (**b**).

It is well known that the phytoremediation capacity represents the ability of a plant to remove toxic compounds from the environment [82,83,84,85]. In the present study, the phytoremediation capacity of the analyzed mushrooms was assessed by using the transfer factor (also known as bioaccumulation or bioconcentration factor), which represents the capacity to absorb one or more heavy metals from soil. The obtained results are presented in Figure 4 and Table 11 (as well as in the Supplementary Material S2—Mushroom samples).

The mean values of the transfer factor for *A. mellea* show a good phytoremediation capacity for all analyzed HMs (mean values 14.559—34.701%). At the same time, the maximum value for cadmium accumulation was recorded in the *M. procera* cap subsample (76.774%), which is another mushroom species with good phytoremediation ability. The lowest levels of *TF* were recorded in the *Agaricus* samples (*A. bisporus* and *A. campestris*).

The highest *TF* value for chromium was recorded in the cap subsample C18 of *M. procera* (115.648%), while the highest *TF* value for cadmium was recorded in the cap subsample C19 of *M. procera* (76.774%), which means that this sample can be considered a hyper-accumulator of these metals and can be successfully used in the phytoremediation of contaminated soils. In almost all cases, the cap subsample recorded a higher *TF* than the stipe subsample; these results are closely correlated with the reported data of other authors [86,87,88,89] due to the increased physiological activity in these parts of the fruiting body [90].

### 3.5. Health Risk Assessment

The estimated daily intake (*EDI*) represents the amount of metal to be taken into the body daily depending on the consumption of the mushroom species investigated in this research. The obtained results compared with recommended values or tolerable intake level and the carcinogenic risk induced by Pb exposure are presented in Table 12 and Supplementary Material S2—Mushroom samples.

The obtained *EDI* values for adults are higher than the values for children due to the recommended amount of mushrooms per day, for each age category. Taking into account the tolerable intake levels or the recommended daily intake values, it can be assumed that three of the analyzed species (i.e., *A. campestris*, *A. mellea*, and *M. procera*) induce high cadmium daily intake for children, but for the adult category, all analyzed species induce high cadmium daily intake. It is well known and published in previous studies that oral cadmium exposure induces bone (i.e., osteoporosis, fractures) [91,92,93], cancer [94], cardiovascular [95], endocrine [96], kidney [97], and reproductive diseases [98], as well as hyperuricemia [99], anemia [100], or neurodevelopment issues [101]. In the case of chromium, four species (i.e., *A. bisporus*, *A. mellea*, *M. excoriata*, and *M. procera*) were recorded with values higher than the recommended daily intake when it comes to the children’s category and, similar to cadmium, all analyzed species induce high chromium daily intake. The main health effects of ingested chromium (i.e., trivalent and hexavalent) have already been studied: hepatotoxicity [102,103], neurotoxicity [102,104], nephrotoxicity [102,105], immunotoxicity [102,106], and developmental toxicity [102,107]. In terms of collecting sites, Ploiesti City records cadmium values higher than the tolerable intake level for children and of all studied cities for the adult category, while in the case of chromium, Sites 1, 2, and 6 recorded values higher than the recommended daily intake for children and all studied cities for the adult category. Even if the lead values do not exceed the tolerable intake level, the carcinogenic risk induced by the lead content shows higher values than the acceptable risk level for all mushroom species and for all studied cities, in the case of both age categories.

The estimated daily intake (*EDI*) represents the first step in the assessment of the health risk; the second step is represented by the daily intake of metals (*DIM*); the obtained values are presented in Table 13 and Supplementary Material S2—Mushroom samples.

The evaluation of the daily metal intake data was made taking into account the values of *EDI* reported to the body mass for each age category. The mean values were calculated in terms of both species and collecting sites (Table 13 and Supplementary Material S2—Mushroom samples). The mean *DIM* values for Cd, Cr, Cu, Ni, and Pb were found to be 0.0010, 0.0013, 0.0043, 0.0018, and 0.0020 for adults and 0.0015, 0.0019, 0.0065, 0.0027, and 0.0030 for children. Regarding the mushroom species, *M. procera* recorded the maximum values for cadmium and chromium and *A. mellea* recorded the maximum values for copper and lead, while for nickel, *B. edulis* recorded the maximum value for both age categories. In terms of collecting sites, Ploiesti City has the maximum value for cadmium, Campina City for copper, Sinaia City for nickel, and Azuga City for chromium and lead—these observations are for both age categories. Comparative with *EDI* values, the *DIM* data were higher for children than for adults due to the body mass taken into account.

Globally, normal values for copper concentration in unpolluted soils range from 2 to 109 mg/kg. However, anthropogenic activities such as mining, oil refining, waste incineration, fossil fuel burning, road traffic, and the widespread use of agrochemicals (such as fungicides, fertilizers, pesticides, and herbicides), as well as soil amendments, have led to an increase in the level of copper in the environment [108]. Copper is an essential element for humans and plants when present in small amounts, but in excessive amounts, it can have harmful effects. Copper concentrations in dry biomass between 20 and 100 mg/kg are considered toxic to most plants [109]. The Acceptable Daily Intake (ADI) for copper has been reduced from 0.15 mg/kg body weight to 0.07 mg/kg body weight by the European Food Safety Authority [110]. This reduction reflects the need to maintain copper concentrations in food at safe levels, as an amount exceeding this level can be toxic. EFSA has determined that, in the case of an adult weighing 70 kg, an amount greater than 10 mg/day of copper can have major harmful effects on the body. In addition, the World Health Organization recommends a maximum daily intake of 0.5 mg of copper per kilogram of body weight for adults (which would equate to approximately 35 mg of copper per day for a 70 kg adult) [111]. On the other hand, the United States Environmental Protection Agency has established a reference level for daily copper intake of 0.04 mg per kilogram of body weight per day (for a 70 kg adult, which would equate to approximately 2.8 mg per day) [112]. Copper is not very mobile in soil and tends to accumulate at the soil surface due to its specific adsorption on mineral and organic fractions. Average concentrations of copper in the topsoil are about 55 mg/kg. Generally, the average concentration of copper in soil ranges from 5 to 70 mg/kg in protected areas and is higher in soils located near smelters and mining areas [113].

For plants, including mushrooms, it is the micronutrient considered vital for growth and development, a structural constituent of numerous regulatory proteins, conferring key roles in CO_2_ assimilation, by participating in mitochondrial respiration, cell wall metabolism, photosynthetic electron transport, responses to oxidative stress, protein synthesis, and so on. In general, a content of 5–30 mg/kg Cu is considered satisfactory for plant tissues [114]. On the other hand, concentration beyond the critical limits promotes leaf chlorosis, causing cytotoxicity. For example, the recommended value in food crops for copper is 30 mg kg^−1^ [115]. Chen et al. revealed that an excessive amount of copper interferes with nutrient absorption, inhibits the photosynthesis of plants, and also affects the functions of key cellular components such as lipids, ARD, or DNA [116]. Due to its dual nature (essential at an optimal level but toxic at high levels), this metal involves a complex network of uptake, sequestration and transport, essentiality and toxicity, and detoxification within plants as already highlighted by Shabbir et al. in their research [114].

One of the most significant soil pollutants is lead, which, once in the soil, can be easily transferred to groundwater, or surface water; it can be absorbed by plants, translocated to their various components, and then transferred to humans, affecting the entire food chain. In soil, this heavy metal is mostly retained by clay loams and extremely fine particles of organic matter. On the other hand, the availability of lead ions in soil depends on its solubility. At a low soil pH (pH < 5), lead ions are retained less and are more soluble, and at a higher pH (pH > 6.5), lead ions are retained more strongly, and their solubility is lower [117]. In previous researches, it was revealed that lead influences the photosynthesis of plants, by inhibiting the activity of carboxylation enzymes, with the specification that a high concentration of Pb^2+^ causes inhibition of enzyme activities, water imbalance, changes in cell membrane permeability, and mineral nutrition disturbances in plants [31,40]. In addition, Pb^2+^ inhibits the activity of enzymes at the cellular level through its reaction with the sulfhydryl groups of plants. Along with Co, Cr, Ni, and Cd, Pb is considered an “environmental health hazard”, and was included in the priority list of dangerous substances, in the first ten positions, by the Agency for the Register of Toxic Substances and Medicines [118].

Lead, being a very toxic metal, can damage the nervous system and kidneys of children. Young children are especially susceptible to lead poisoning because they have 4–5 times greater intestinal absorption. In adults, at blood lead levels of 50–80 μg·dL^−1^, signs of chronic lead toxicity may occur, including fatigue, insomnia, irritability, headache, joint pain, and gastrointestinal symptoms. The total amount of Pb^2+^ for an adult can vary from 20 to 150 μg·day^−1^ in most countries, with a daily dose of 0.2–2 mg being considered safe. The main routes of entry of lead into the human body, i.e., ingestion and inhalation, involve the intestinal system. Lead exposure causes 0.6% of all global diseases [119]. The accumulation of lead in biological substrates is determined by the level of exposure to lead toxicity. Thus, if lead ions are found in blood samples, then a recent exposure is evidenced; if lead ions are present in the teeth, it means that the exposure was long-term. Lead ions present in the bones show an accumulation of metal ions during the fetal period, and the presence of lead ions in hair is an indicator of exposure for a long period.

Nickel (Ni), along with lead (Pb), is one of the most toxic environmental pollutants. Ni can enter the human body through inhalation, ingestion, and dermal absorption and is known to cause a variety of adverse health effects, including contact dermatitis, pulmonary fibrosis, cardiovascular, and kidney disease [120,121]. There is also evidence that some nickel compounds are carcinogenic and have been linked to lung and respiratory tract cancer [122]. Regarding inhalation, the particle size of particulate matter (PMs) is a determining factor in Pb deposition in the body [123]. Inhalation exposure in occupational settings is a major pathway for nickel-induced toxicity in the respiratory system, lungs, and immune system [124]. Water-soluble nickel compounds are absorbed into the body through the lungs by diffusion, while water-insoluble nickel compounds enter the respiratory system by phagocytosis and remain in the lungs for a longer period. Guo et al., (2019) reported that nickel ingested or inhaled is accumulated in the kidneys, which are the main target organs for deposition, followed by the brain and pancreas [125]. In brief, Ni as a metal is immunotoxic, hemotoxic, neurotoxic, genotoxic, nephrotoxic, and hepatotoxic. The International Agency for Research on Cancer (IARC), in 1990, classified all nickel compounds, except for metallic nickel, to be carcinogenic to humans [126,127]. The carcinogenic potential of nickel compounds depends on their solubility in water. Depending on the dose and duration of exposure, as an immunotoxic and carcinogenic agent, Ni can cause a variety of health effects, such as contact dermatitis, cardiovascular disease, asthma, pulmonary fibrosis, and respiratory tract cancer. Apart from those mentioned, in biological systems, Ni plays a well-established role. For instance, nickel deficiency in the human body leads to abnormal cellular morphology and oxidative metabolism, increases or decreases lipid levels, and can induce anemia in terms of reduced iron adsorption [121,126,127]. Several studies revealed that the average daily intake of nickel from various fresh or cooked foods (i.e., vegetables, fruits, grains, etc.), water, and beverages is 101–162 μg per day for adults, specifically, 136–140 μg per day for males, and 107–109 μg per day for females. The average nickel intakes for different children’s categories were recorded, such as for those aged 0–6 months, a value of 9 μg per day; for those aged 7–12 months, a value of 39 μg per day; for ages 1–3 years, 82 μg per day; and for ages 4–8 years, 99 μg per day. In addition, the average nickel intake for pregnant women was revealed to be 121 μg per day [121,128,129].

Chromium (Cr) is one of the most significant environmental and human contaminants. The most stable and toxic forms of Cr, depending on pH, are Cr(III) (less toxic) and Cr(VI) (highly toxic) [130]. Thus, Cr(III) is a microelement with relatively low toxicity and is only slightly soluble in water, while Cr(VI) is known to be carcinogenic, quite water-soluble, and mobile [131]. He and Li (2020) revealed that Cr(VI) risks to human health depend on the dose, exposure level, and duration [132]. Long-term and continuous exposure to chromium even at low concentrations, i.e., in the case of occupational exposure, can affect the skin, eyes, blood, respiratory, and immune systems [133]. Long-term exposure to Cr(VI) can cause acute diseases and adverse reactions, such as allergic reactions and skin ulcers, and even lead to the well-known triad of mutagenesis, carcinogenesis, and teratogenesis [134]. The World Health Organization (WHO) suggests 50 µg/L of Cr as a guideline value based on health for drinking water [135]. Most wastewater contains Cr(VI) and organic pollutants, and many studies revealed the synergy between them in photocatalytic degradation. Therefore, the toxicity and mobility of Cr is mainly controlled by its oxidation state and, thus, by redox reactions [136].

Of all the heavy metals, cadmium (Cd) is one of the most mobile and potentially toxic elements (PTEs) that have garnered attention due to their persistence, toxicity, non-degradability, and bioaccumulation [29,137,138]. Even at low concentrations, it is toxic to all life forms, including plants, fish, birds, mammals, and microorganisms [139]. In brief, Cd as an environmental pollutant ranked eighth place in the Top 20 priority hazardous substances due to its high toxicity and slow rate of metabolism. Cadmium is mainly bound to blood cells and only after chronic exposure is it also stored in the kidney cortex. Therefore, blood cadmium levels represent recent exposure to cadmium, while urine levels of cadmium are more likely to be a measure of chronic and cumulative exposure over a lifetime [140]. The health effects of cadmium exposure are exacerbated by the inability of the human body to excrete cadmium [141]. Cd absorption occurs primarily through the respiratory system and to a lesser extent through the gastrointestinal system, while dermal absorption is relatively rare. When cadmium enters the body, it is transported into the blood via erythrocytes and albumin and then accumulates in the kidneys, liver, and intestine. In humans, Cd exposure can lead to a variety of adverse effects, such as renal and hepatic dysfunction, pulmonary edema, and damage to the adrenal and hematopoietic systems. An association between markers of Cd exposure (blood and urine) and coronary heart disease, stroke, peripheral arterial disease, and atherogenic changes in the lipid profile has also been observed by Vogel et al. [140]. In addition to its cytotoxic effects that could lead to apoptotic or necrotic events, cadmium is a proven human carcinogen (group I of the International Agency for Research on Cancer classification) [140]. Cadmium is statistically associated with an increased risk of cancer and participates in the process of bone demineralization [141]. Some conflicting reports are suggesting that cadmium may be an anti-inflammatory factor [142].

The last step in the health risk assessment is the calculation of the health risk index (*HRI*) based on the *DIM* values and oral reference doses (*RfD*); the obtained results are presented in Table 14 and Supplementary Material S2—Mushroom samples.

As a result of the calculations, it was highlighted that *M. procera* harms human health in terms of cadmium and chromium, as well as the total hazard quotient; *A. mellea* has a negative effect on human health in terms of copper and lead; and *B. edulis* has a negative effect on human health in terms of nickel—for both age categories (i.e., adults and children). The worrying data were related to cadmium because in the case of all species (except *A. bisporus*), the HRI exceeded the recommended level 1 for the children’s category; for the adult category, just *A. campestris*, *A. mellea*, and *M. procera* exceeded this level. The classification of cities according to the maximum *HRI* value is identical to that presented in the case of *DIM*: Ploiesti City has the maximum value for cadmium, Campina City for copper, Sinaia City for nickel, and Azuga City for chromium and lead—these observations are for both age categories. Similar data were obtained for *B. edulis* and *A. campestris* collected from Salaj County (North-Western Romania) [143] and on *M. procera*, *B. edulis*, and *A. campestris* collected from Dambovita County (South-Eastern Romania) [144], which demonstrate the human risk induced by the consumption of wild edible mushrooms collected from the uncontrolled environment.

### 3.6. Statistical Analysis of Obtained Results

Within the analyzed soil samples, the descriptive values of the indicators presented in Table 15 reveal a significant variability.

For cadmium, the identified concentrations vary between a minimum value of 2.324 and a maximum value of 6.416, which correspond to a variation interval of 4.092 in the case of the studied samples. In contrast, copper concentrations highlight a considerably larger variation interval, of approximately 32.166, determined by the difference between the minimum value recorded (11.804) and the maximum value (43.970). This high variability can be mainly attributed to the heterogeneous location of the soil samples, which indicates a significant degree of heterogeneity of environmental factors and pollution sources in the investigated areas.

Following the data obtained on mushroom samples the relevant variables are presented in Table 16.

A notable aspect is the increased homogeneity of these samples. The analysis revealed that the variation ranges for the five elements analyzed are significantly lower in mushroom samples than in soil. Thus, the variation ranges identified are 1.607 for cadmium, 1.727 for chromium, 2.314 for nickel, 3.027 for lead, and 3.929 for copper. This relatively high homogeneity can be explained by the influence of the collected species and by the fact that the variability of the analyzed locations affects the soil to a greater extent than the biological matrix of the mushrooms. This finding emphasizes the moderating role of local conditions on the chemical composition of the final product.

The interpretation of the Pearson correlations (Table 17) is based on the analysis of the significance and the values of the correlation coefficient (r). In the context of the information presented above, the values indicate the strength and direction of the linear relationships between the different metals (Cd, Cr, Cu, Ni, and Pb) determined in the fungal and soil samples. Significant relationships exist between most elements, but the variability suggests differences in the sources or accumulation mechanisms in the studied samples. Pb shows strongly significant correlations with Cd (r = 0.864, *p* < 0.01), Cr (r = 0.820, *p* < 0.01), Cu (r = 0.854, *p* < 0.01), and Ni (r = 0.918, *p* < 0.01).

**Figure 5:**
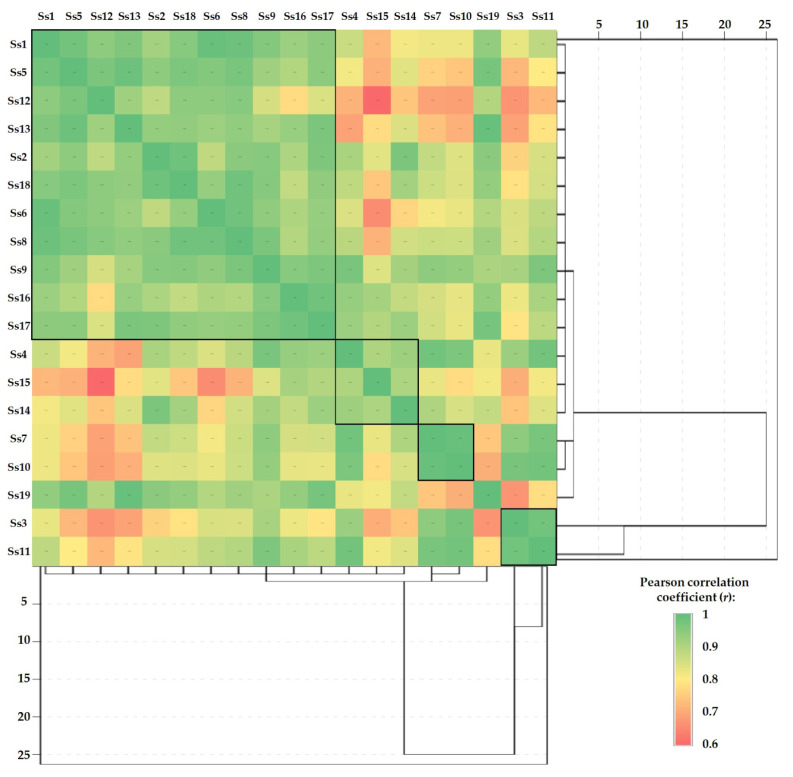
Pearson correlations between the soil samples in terms of HMs content combined with hierarchical cluster analysis.

**Figure 6:**
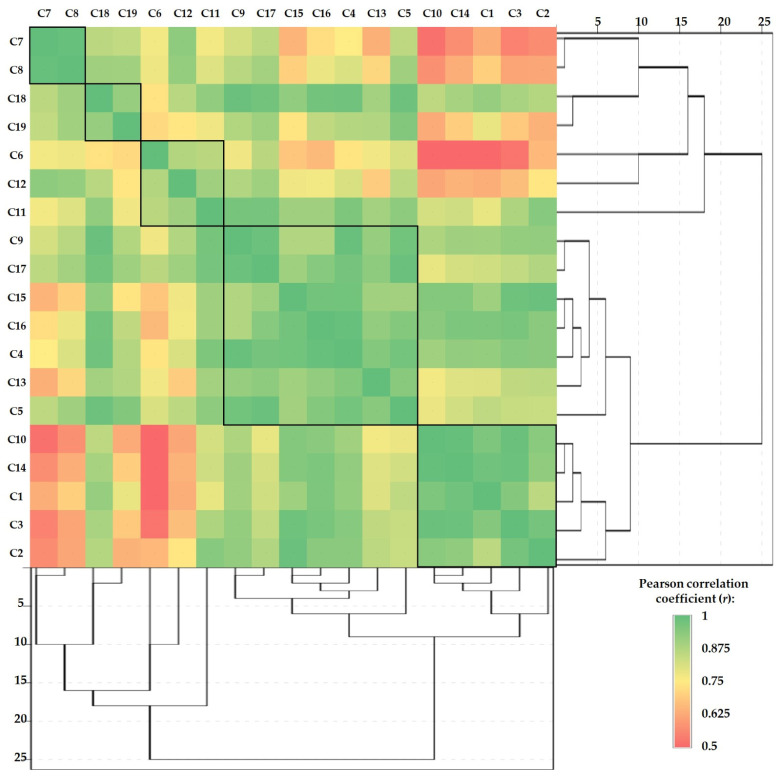
Pearson correlations between the mushroom cap subsamples in terms of HMs content combined with hierarchical cluster analysis.

**Figure 7:**
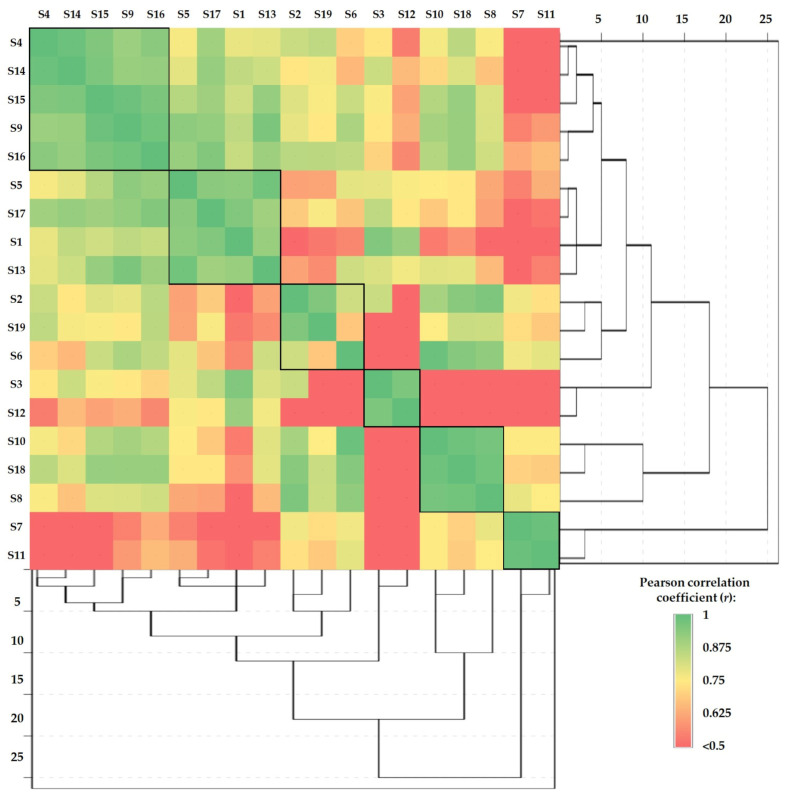
Pearson correlations between the mushroom stipe subsamples in terms of HMs content combined with hierarchical cluster analysis.

The Pearson correlations between samples (made separately for each sample) combined with hierarchical cluster analysis are presented in Figure 5, Figure 6 and Figure 7. This type of graph is very useful to identify the similarities between samples and to determine how closely two or more variables are related.

The results of the interdependence between the analyzed soil samples, as reflected by the Pearson correlations and the hierarchical clusters presented graphically in Figure 5, show the formation of three sample groups with similar characteristics. Among these, the majority cluster characterized by positive correlations with r ranging from 0.776 to 0.993 was observed, formed by samples Ss1, Ss2, Ss5, Ss6, Ss8, Ss9, Ss12, Ss13, and Ss16–Ss18. Moreover, two other minor clusters formed by samples Ss3 and Ss11 (r = 0.982), and by Ss 7 and Ss10 (r = 0.993), respectively, are distinguished. At the same time, minor similarities were observed in the case of the cluster formed by Ss4-Ss14-Ss15 samples (r = 0.904 ÷ 0.926).

Regarding the studied mushroom cap subsamples, the graph obtained based on Pearson correlations and cluster analysis (Figure 6) shows the formation of two major and three minor clusters, as follows: C4-C5-C9-C13-C15-C16-C17 (r = 0.900 ÷ 0.995), C1-C2-C3-C10-C14 (r = 0.886 ÷ 0.996), C7-C8 (r = 0.995), C18-C19 (r = 0.935), and C6-C11-C12 (r = 0.889÷0.923), respectively.

In the case of the studied mushroom stipe subsamples, the Pearson correlations combined with cluster analysis (Figure 7) highlight the formation of two major and four minor clusters, as follows: S4-S9-S14-S15-S16 (r = 0.909÷0.987), S1-S5-S13-S17 (r = 0.902÷0.953), S2-S6-S19 (r = 0.684÷0.955), S3-S12 (r = 0.959), S8-S10-S18 (r = 0.971÷0.983), and S7-S11 (r = 0.988), respectively.

## 4. Conclusions

Currently, soil pollution has become one of the most important and debated issues. The presence of heavy metals in the soil is closely related to the deterioration of its quality and, implicitly, of the quality of life, thus justifying concerns in the direction of reducing their impact. This study revealed that the bioaccumulation of heavy metals in mushrooms and the human body as well, over time, can lead to an increase in their concentration, compared to the metal concentration in the soil (i.e., habitat of plants). This aspect is explained by the fact that heavy metal compounds accumulate in living organisms (i.e., fungi and humans) when they are assimilated and stored at a higher rate than are metabolized or eliminated. The toxicity of a heavy metal clearly depends on its oxidation state, which involves higher mobility in soil under acidic conditions, facilitating their transport and spread in the environment and living organisms. In conclusion, the issue of the essentiality and/or toxicity of metals become more complex when their applied/predominant levels are considered, together with different soil conditions and fungal species. Therefore, the elucidation of the biochemical behavior of the metals studied in the soil–mushroom–human systems is extremely important, especially in the case of the two categories, children and the elderly, due to severe disorders ranging from anemia to liver illness, digestive, neurological, Alzheimer’s, or tumor diseases.

Taking into account the tendency of heavy metals to bioaccumulate in the soil and implicitly in mushrooms with direct transfer into the human body (by ingestion, inhalation, or dermal exposure), and the serious consequences on the health of all age groups, this comprehensive study is the first scientific incursion with a direct alert on the population of areas with touristic potential raised from the protected natural area according to national and European legislation. On the other hand, these findings are useful for policymakers, contributing to increasing public awareness about the risk of consumption of mushrooms collected from different protected areas, even though these can be considered safe. Lastly, the findings of this study will provide useful directions for future studies in terms of the nutritional benefits of mushrooms related to health.

The analytical technique (ICP-MS) and tools used to assess the health effects of soil and wild edible mushrooms are presented in this study. These include determination of HMs (i.e., Cd, Cr, Cu, Ni, and Pb) in soil samples, index of geo-accumulation (I_geo_), contamination factor (CF), pollution loading index (PLI), chronic daily dose calculated for three exposure pathways (ingestion, dermal, and inhalation) as well as hazard quotient of non-cancer risks (HQ and HQ_total_) and the carcinogenic risks (CR and HI_total_). In terms of mushroom samples, HMs content was determined by the same analytical method (ICP-MS) and the calculations were made for the phytoremediation capacity (transfer factor (TF) for cap and stipe subsamples) and health risk assessment: estimated daily intake (EDI), carcinogenic risk induced by the lead content (CR_Pb_), daily intake metals (DIM), and health risk index (HRI). The exposure assessment was calculated for adults and children in the case of both sample categories (i.e., soil and mushroom). This study implies, also, the mean values in terms of species and collecting sites for all data.

The obtained results for HMs in soil samples were compared with recommended values and European average values, highlighting Cd values that exceed the maximum threshold established by the Romanian Order no. 756/1997 [63] and are 40 times higher than the European average value [61]. In the case of Cu, the results exceed the European average value in five collection sites, while for the other HMs (i.e., Cr, Ni, and Pb) the values are under the normal threshold established by the Romanian Order no. 756/1997 and the European average value. The high values of Cd induced high values of I_geo_ (i.e., >6.0), which include these soils in the extremely polluted class, and high values of CF (i.e., >3.0), which indicate a moderate to considerable contamination. In terms of exposure assessment, the chronic daily dose was found in order of ingestion > dermal > inhalation exposure. The values for children were 10 times higher than for adults, demonstrating that children are more exposed than adults. The same ratio between children and adults was maintained in the case of hazard quotient of non-cancer risks and hazard index of cancer risks.

The obtained results for HMs in mushroom samples (including cap and stipe subsamples) were compared with the maximum admitted limits established by the Romanian Order no. 975/1998 (i.e., fresh vegetable category), the Commission Regulation (EC) no. 2023/915 (i.e., Brassicaceae vegetables and wild mushrooms categories), and Codex Alimentarius CODEX-STAN 193-1995 (i.e., Brassicaceae vegetables). Based on these values, the phytoremediation capacity of mushrooms was determined using the transfer factor for both subsamples (i.e., cap and stipe); the highest values were recorded in cap subsamples of *M. procera* for Cr (115.648%) and stipe subsamples of S14 of *B. edulis* also for Cr (69.880%). The estimated daily intake values highlight that three of the analyzed species (i.e., *A. campestris*, *A. mellea*, and *M. procera*) induce high cadmium daily intake for children, but for the adult category, all analyzed species induce high cadmium daily intake. In the case of daily intake metals, *M. procera* recorded the maximum values for cadmium and chromium and *A. mellea* recorded the maximum values for copper and lead, while for nickel, *B. edulis* recorded the maximum value for both age categories. The worrying results of this study are related to cadmium, because in all species (except *A. bisporus*), the HRI exceeded the recommended level 1 for the children’s category; for the adult category, just *A. campestris*, *A. mellea*, and *M. procera* exceeded this level.

The statistical processing of the obtained data is characterized by a high degree of novelty due to the Pearson correlations between the samples/subsamples in terms of HMs content combined with hierarchical cluster analysis. This type of analysis is very useful in identifying the similarities between samples and in determining how closely two or more variables (in this case samples) are related.

In Romania, wild mushrooms are considered as delicacies and people consume mushrooms from the analyzed species more often than they consume mushrooms grown in controlled environments. More than that, if the mushrooms are harvested from forests that are in or near the areas protected by law (i.e., natural parks, reservations, etc.), people consider those mushrooms safer for consumption than those sold in markets. The authors present these results as an alarm signal for citizens and authorities in order to prevent people from harvesting and consuming those types of mushrooms.

## Figures and Tables

**Table 1:** Information about the collected and analyzed six wild edible indigenous mushroom species such as location, collection season, subsamples, and substrate codes.

No.	Mushroom Specie	Localization	Collection Period	Subsamples (Codes)	Substrate (Codes)
1	*Agaricus bisporus*	Azuga/Site 6	Summer 2023	Cap (C1) and stipe (S1)	Soil (Ss1)
2	*Agaricus bisporus*	Busteni/Site 5	Summer 2023	Cap (C2) and stipe (S2)	Soil (Ss2)
3	*Agaricus bisporus*	Comarnic/Site 3	Summer 2023	Cap (C3) and stipe (S3)	Soil (Ss3)
4	*Agaricus bisporus*	Sinaia/Site 4	Summer 2023	Cap (C4) and stipe (S4)	Soil (Ss4)
5	*Agaricus campestris*	Campina/Site 2	Summer 2023	Cap (C5) and stipe (S5)	Soil (Ss5)
6	*Agaricus campestris*	Ploiesti/Site 1	Summer 2023	Cap (C6) and stipe (S6)	Soil (Ss6)
7	*Armillaria mellea*	Azuga/Site 6	Autumn 2023	Cap (C7) and stipe (S7)	Soil (Ss7)
8	*Armillaria mellea*	Busteni/Site 5	Autumn 2023	Cap (C8) and stipe (S8)	Soil (Ss8)
9	*Armillaria mellea*	Campina/Site 2	Autumn 2023	Cap (C9) and stipe (S9)	Soil (Ss9)
10	*Armillaria mellea*	Comarnic/Site 3	Autumn 2023	Cap (C10) and stipe (S10)	Soil (Ss10)
11	*Armillaria mellea*	Ploiesti/Site 1	Autumn 2023	Cap (C11) and stipe (S11)	Soil (Ss11)
12	*Armillaria mellea*	Sinaia/Site 4	Autumn 2023	Cap (C12) and stipe (S12)	Soil (Ss12)
13	*Boletus edulis*	Busteni/Site 5	Autumn 2023	Cap (C13) and stipe (S13)	Soil (Ss13)
14	*Boletus edulis*	Comarnic/Site 3	Autumn 2023	Cap (C14) and stipe (S14)	Soil (Ss14)
15	*Boletus edulis*	Sinaia/Site 4	Autumn 2023	Cap (C15) and stipe (S15)	Soil (Ss15)
16	*Macrolepiota excoriata*	Campina/Site 2	Autumn 2023	Cap (C16) and stipe (S16)	Soil (Ss16)
17	*Macrolepiota excoriata*	Ploiesti/Site 1	Autumn 2023	Cap (C17) and stipe (S17)	Soil (Ss17)
18	*Macrolepiota procera*	Campina/Site 2	Summer 2023	Cap (C18) and stipe (S18)	Soil (Ss18)
19	*Macrolepiota procera*	Ploiesti/Site 1	Summer 2023	Cap (C19) and stipe (S19)	Soil (Ss19)

**Table 2:** Samples digestion process using TOPwave microwave-assisted pressure digestion system (Analytik Jena, Jena, Germany).

Sample Type	Mixture	Waiting Time	Digestion Program			Cooling Time	Volumetric Flask
Mushroom	~0.4 g sample 6 mL HNO_3_ (65%) 1 mL H_2_O_2_ (30%)	20 min	Step	1	2	30 min	25 mL
			T [°C]	170	200		
			P [bar]	40	40		
			Power [%]	80	90		
			Ramp [min]	5	1		
			Time [min]	10	15		
Soil	~4 g sample 2.5 mL HNO_3_ (65%) 7.5 mL HCl (37%)	30 min	Step	1		30 min	50 mL
			T [°C]	140			
			P [bar]	40			
			Power [%]	90			
			Ramp [min]	8			
			Time [min]	15			

**Table 3:** Analytical results of the elements in NIST SRM 2710a and SRM 1515, as well as the LOD and LOQ values.

SRM [mg·kg^−1^]	Cd	Cr	Cu	Ni	Pb
Standard Materials (certified, reference * or information mass fraction ** values)					
SRM 2710a—certified	12.300 ± 0.300	23.000 ± 6.000 *	0.342 ± 0.005	8.000 ± 1.000 *	0.552 ± 0.003
SRM 2710a—measured	12.577 ± 0.687	21.529 ± 1.810	0.317 ± 0.033	7.187 ± 0.661	0.496 ± 0.037
% recovery SRM	102	94	93	90	90
SRM 1515—certified	0.0132 ± 0.0015	0.3 **	5.690 ± 0.130	0.936 ± 0.094	0.470 ± 0.024
SRM 1515—measured	0.014 ± 0.002	0.344 ± 0.029	6.013 ± 0.055	0.896 ± 0.077	0.503 ± 0.048
% recovery SRM	106	115	106	96	107
Limit of detection (LOD)/Limit of quantitation (LOQ)					
LOD [μg/kg]	0.133	1.489	1.202	0.669	1.223
LOQ [μg/kg]	0.148	2.048	1.496	0.802	1.305

**Table 7:** Mean values obtained for the index of geo-accumulation (*I_geo_*) of soils, in terms of both species and collecting sites.

	Cd	Cr	Cu	Ni	Pb
Collecting sites					
Site 1	6.105	−2.114	1.605	−1.440	−0.172
Site 2	6.380	−2.347	1.124	−1.704	−0.445
Site 3	6.531	−2.821	1.657	−2.155	−0.485
Site 4	6.242	−2.068	1.050	−2.060	−0.460
Site 5	6.251	−2.536	1.141	−1.588	−0.361
Site 6	6.603	−2.292	1.199	−2.081	−0.603
Mushroom species					
*Agaricus bisporus* (n = 24)	6.500	−2.310	1.403	−1.903	−0.553
*Agaricus campestris* (n = 12)	6.493	−2.079	1.133	−1.443	−0.558
*Armillaria mellea* (n = 36)	6.637	−2.415	1.581	−1.738	−0.350
*Boletus edulis* (n = 18)	5.820	−2.445	1.044	−2.142	−0.253
*Macrolepiota excoriata* (n = 12)	6.030	−1.802	0.981	−1.901	−0.494
*Macrolepiota procera* (n = 12)	5.945	−2.941	1.197	−1.498	−0.208

**Table 8:** Chronic daily dose (expressed as mg·kg^−1^·day^−1^) of HMs in the soil calculated for adults and children.

Path	Site	Adults					Children				
		Cd	Cr	Cu	Ni	Pb	Cd	Cr	Cu	Ni	Pb
Ingestion	Site 1	5.43 × 10^−6^	5.46 × 10^−6^	3.04 × 10^−5^	1.61 × 10^−5^	1.79 × 10^−5^	5.07 × 10^−5^	5.10 × 10^−5^	2.83 × 10^−4^	1.50 × 10^−4^	1.67 × 10^−4^
	Site 2	6.27 × 10^−6^	5.24 × 10^−6^	1.88 × 10^−5^	1.32 × 10^−5^	1.42 × 10^−5^	5.85 × 10^−5^	4.89 × 10^−5^	1.76 × 10^−4^	1.24 × 10^−4^	1.32 × 10^−4^
	Site 3	7.12 × 10^−6^	3.62 × 10^−6^	2.96 × 10^−5^	9.77 × 10^−6^	1.39 × 10^−5^	6.65 × 10^−5^	3.38 × 10^−5^	2.76 × 10^−4^	9.12 × 10^−5^	1.30 × 10^−4^
	Site 4	5.94 × 10^−6^	5.90 × 10^−6^	1.78 × 10^−5^	1.10 × 10^−5^	1.42 × 10^−5^	5.54 × 10^−5^	5.50 × 10^−5^	1.66 × 10^−4^	1.03 × 10^−4^	1.32 × 10^−4^
	Site 5	5.93 × 10^−6^	4.10 × 10^−6^	1.90 × 10^−5^	1.43 × 10^−5^	1.50 × 10^−5^	5.53 × 10^−5^	3.83 × 10^−5^	1.77 × 10^−4^	1.34 × 10^−4^	1.40 × 10^−4^
	Site 6	7.29 × 10^−6^	5.16 × 10^−6^	1.99 × 10^−5^	1.07 × 10^−5^	1.27 × 10^−5^	6.81 × 10^−5^	4.82 × 10^−5^	1.85 × 10^−4^	9.97 × 10^−5^	1.19 × 10^−4^
Inhalation	Site 1	7.98 × 10^−10^	8.03 × 10^−10^	4.46 × 10^−9^	2.37 × 10^−9^	2.63 × 10^−9^	1.42 × 10^−9^	1.42 × 10^−9^	7.92 × 10^−9^	4.19 × 10^−9^	4.67 × 10^−9^
	Site 2	9.21 × 10^−10^	7.70 × 10^−10^	2.77 × 10^−9^	1.95 × 10^−9^	2.09 × 10^−9^	1.63 × 10^−9^	1.37 × 10^−9^	4.91 × 10^−9^	3.45 × 10^−9^	3.70 × 10^−9^
	Site 3	1.05 × 10^−9^	5.32 × 10^−10^	4.35 × 10^−9^	1.44 × 10^−9^	2.05 × 10^−9^	1.86 × 10^−9^	9.43 × 10^−10^	7.72 × 10^−9^	2.55 × 10^−9^	3.63 × 10^−9^
	Site 4	8.73 × 10^−10^	8.67 × 10^−10^	2.61 × 10^−9^	1.62 × 10^−9^	2.09 × 10^−9^	1.55 × 10^−9^	1.54 × 10^−9^	4.63 × 10^−9^	2.88 × 10^−9^	3.70 × 10^−9^
	Site 5	8.72 × 10^−10^	6.03 × 10^−10^	2.79 × 10^−9^	2.11 × 10^−9^	2.21 × 10^−9^	1.55 × 10^−9^	1.07 × 10^−9^	4.95 × 10^−9^	3.74 × 10^−9^	3.92 × 10^−9^
	Site 6	1.07 × 10^−9^	7.59 × 10^−10^	2.92 × 10^−9^	1.57 × 10^−9^	1.87 × 10^−9^	1.90 × 10^−9^	1.35 × 10^−9^	5.18 × 10^−9^	2.79 × 10^−9^	3.31 × 10^−9^
Dermal	Site 1	9.88 × 10^−9^	9.94 × 10^−9^	5.52 × 10^−8^	2.93 × 10^−8^	3.26 × 10^−8^	5.01 × 10^−8^	5.04 × 10^−8^	2.80 × 10^−7^	1.48 × 10^−7^	1.65 × 10^−7^
	Site 2	1.14 × 10^−8^	9.53 × 10^−9^	3.43 × 10^−8^	2.41 × 10^−8^	2.58 × 10^−8^	5.78 × 10^−8^	4.83 × 10^−8^	1.74 × 10^−7^	1.22 × 10^−7^	1.31 × 10^−7^
	Site 3	1.30 × 10^−8^	6.58 × 10^−9^	5.39 × 10^−8^	1.78 × 10^−8^	2.53 × 10^−8^	6.57 × 10^−8^	3.33 × 10^−8^	2.73 × 10^−7^	9.01 × 10^−8^	1.28 × 10^−7^
	Site 4	1.08 × 10^−8^	1.07 × 10^−8^	3.23 × 10^−8^	2.01 × 10^−8^	2.58 × 10^−8^	5.47 × 10^−8^	5.44 × 10^−8^	1.64 × 10^−7^	1.02 × 10^−7^	1.31 × 10^−7^
	Site 5	1.08 × 10^−8^	7.46 × 10^−9^	3.46 × 10^−8^	2.61 × 10^−8^	2.74 × 10^−8^	5.47 × 10^−8^	3.78 × 10^−8^	1.75 × 10^−7^	1.32 × 10^−7^	1.39 × 10^−7^
	Site 6	1.33 × 10^−8^	9.40 × 10^−9^	3.61 × 10^−8^	1.94 × 10^−8^	2.31 × 10^−8^	6.72 × 10^−8^	4.76 × 10^−8^	1.83 × 10^−7^	9.85 × 10^−8^	1.17 × 10^−7^
Total	Site 1	5.44 × 10^−6^	5.47 × 10^−6^	3.04 × 10^−5^	1.61 × 10^−5^	1.79 × 10^−5^	5.07 × 10^−5^	5.10 × 10^−5^	2.84 × 10^−4^	1.50 × 10^−4^	1.67 × 10^−4^
	Site 2	6.28 × 10^−6^	5.25 × 10^−6^	1.89 × 10^−5^	1.33 × 10^−5^	1.42 × 10^−5^	5.85 × 10^−5^	4.89 × 10^−5^	1.76 × 10^−4^	1.24 × 10^−4^	1.32 × 10^−4^
	Site 3	7.13 × 10^−6^	3.62 × 10^−6^	2.96 × 10^−5^	9.79 × 10^−6^	1.39 × 10^−5^	6.65 × 10^−5^	3.38 × 10^−5^	2.76 × 10^−4^	9.13 × 10^−5^	1.30 × 10^−4^
	Site 4	5.95 × 10^−6^	5.91 × 10^−6^	1.78 × 10^−5^	1.11 × 10^−5^	1.42 × 10^−5^	5.55 × 10^−5^	5.51 × 10^−5^	1.66 × 10^−4^	1.03 × 10^−4^	1.32 × 10^−4^
	Site 5	5.94 × 10^−6^	4.11 × 10^−6^	1.90 × 10^−5^	1.44 × 10^−5^	1.51 × 10^−5^	5.54 × 10^−5^	3.83 × 10^−5^	1.77 × 10^−4^	1.34 × 10^−4^	1.41 × 10^−4^
	Site 6	7.31 × 10^−6^	5.17 × 10^−6^	1.99 × 10^−5^	1.07 × 10^−5^	1.27 × 10^−5^	6.81 × 10^−5^	4.82 × 10^−5^	1.86 × 10^−4^	9.98 × 10^−5^	1.19 × 10^−4^

**Table 9:** Hazard quotient of non-cancer risks and hazard index of cancer risks (the term “total” represents the sum of the three exposure routes: ingestion, inhalation, and dermal) calculated for adults and children, in terms of both species and collecting sites.

		HQ_total_						HI_total_				
Adults		Cd	Cr	Cu	Ni	Pb	Total	Cd	Cr	Ni	Pb	Total
	*A. bisporus*	7.31 × 10^−3^	1.68 × 10^−3^	6.33 × 10^−4^	6.24 × 10^−4^	9.51 × 10^−3^	1.98 × 10^−2^	4.29 × 10^−5^	2.51 × 10^−6^	1.08 × 10^−5^	1.13 × 10^−7^	5.64 × 10^−5^
	*A. campestris*	7.25 × 10^−3^	1.89 × 10^−3^	4.74 × 10^−4^	8.41 × 10^−4^	9.45 × 10^−3^	1.99 × 10^−2^	4.26 × 10^−5^	2.82 × 10^−6^	1.45 × 10^−5^	1.12 × 10^−7^	6.00 × 10^−5^
	*A. mellea*	8.07 × 10^−3^	1.54 × 10^−3^	7.16 × 10^−4^	7.25 × 10^−4^	1.12 × 10^−2^	2.23 × 10^−2^	4.74 × 10^−5^	2.29 × 10^−6^	1.26 × 10^−5^	1.33 × 10^−7^	6.24 × 10^−5^
	*B.edulis*	4.60 × 10^−3^	1.71 × 10^−3^	4.45 × 10^−4^	5.45 × 10^−4^	1.16 × 10^−2^	1.89 × 10^−2^	2.70 × 10^−5^	2.54 × 10^−6^	9.43 × 10^−6^	1.38 × 10^−7^	3.91 × 10^−5^
	*M. excoriata*	5.30 × 10^−3^	2.34 × 10^−3^	4.26 × 10^−4^	6.13 × 10^−4^	9.83 × 10^−3^	1.85 × 10^−2^	3.11 × 10^−5^	3.48 × 10^−6^	1.06 × 10^−5^	1.16 × 10^−7^	4.53 × 10^−5^
	*M. procera*	5.23 × 10^−3^	1.15 × 10^−3^	4.96 × 10^−4^	8.20 × 10^−4^	1.22 × 10^−2^	1.99 × 10^−2^	3.07 × 10^−5^	1.71 × 10^−6^	1.41 × 10^−5^	1.44 × 10^−7^	4.67 × 10^−5^
		Cd	Cr	Cu	Ni	Pb	Total	Cd	Cr	Ni	Pb	Total
	Site 1	5.83 × 10^−3^	1.85 × 10^−3^	7.64 × 10^−4^	8.58 × 10^−4^	1.29 × 10^−2^	2.22 × 10^−2^	3.42 × 10^−5^	2.75 × 10^−6^	1.49 × 10^−5^	1.52 × 10^−7^	5.20 × 10^−5^
	Site 2	6.72 × 10^−3^	1.78 × 10^−3^	4.73 × 10^−4^	7.06 × 10^−4^	1.02 × 10^−2^	1.99 × 10^−2^	3.95 × 10^−5^	2.64 × 10^−6^	1.22 × 10^−5^	1.21 × 10^−7^	5.44 × 10^−5^
	Site 3	7.64 × 10^−3^	1.23 × 10^−3^	7.44 × 10^−4^	5.22 × 10^−4^	9.99 × 10^−3^	2.01 × 10^−2^	4.49 × 10^−5^	1.82 × 10^−6^	9.13 × 10^−6^	1.18 × 10^−7^	5.59 × 10^−5^
	Site 4	6.37 × 10^−3^	2.00 × 10^−3^	4.47 × 10^−4^	5.90 × 10^−4^	1.02 × 10^−2^	1.96 × 10^−2^	3.74 × 10^−5^	2.97 × 10^−6^	1.02 × 10^−5^	1.21 × 10^−7^	5.07 × 10^−5^
	Site 5	6.36 × 10^−3^	1.39 × 10^−3^	4.78 × 10^−4^	7.64 × 10^−4^	1.08 × 10^−2^	1.98 × 10^−2^	3.73 × 10^−5^	2.07 × 10^−6^	1.32 × 10^−5^	1.28 × 10^−7^	5.27 × 10^−5^
	Site 6	7.82 × 10^−3^	1.75 × 10^−3^	5.00 × 10^−4^	5.70 × 10^−4^	9.12 × 10^−3^	1.98 × 10^−2^	4.59 × 10^−5^	2.60 × 10^−6^	9.88 × 10^−6^	1.08 × 10^−7^	5.85 × 10^−5^
Children		Cd	Cr	Cu	Ni	Pb	Total	Cd	Cr	Ni	Pb	Total
	*A. bisporus*	6.61 × 10^−2^	1.55 × 10^−2^	5.89 × 10^−3^	5.65 × 10^−3^	8.86 × 10^−2^	1.82 × 10^−1^	4.00 × 10^−4^	2.33 × 10^−5^	1.00 × 10^−4^	1.05 × 10^−6^	5.25 × 10^−4^
	*A. campestris*	6.55 × 10^−2^	1.74 × 10^−2^	4.42 × 10^−3^	7.62 × 10^−3^	8.80 × 10^−2^	1.83 × 10^−1^	3.97 × 10^−4^	2.62 × 10^−5^	1.35 × 10^−4^	1.04 × 10^−6^	5.59 × 10^−4^
	*A. mellea*	7.29 × 10^−2^	1.42 × 10^−2^	6.66 × 10^−3^	6.57 × 10^−3^	1.05 × 10^−1^	2.05 × 10^−1^	4.42 × 10^−4^	2.13 × 10^−5^	1.16 × 10^−4^	1.24 × 10^−6^	5.81 × 10^−4^
	*B.edulis*	4.16 × 10^−2^	1.57 × 10^−2^	4.14 × 10^−3^	4.94 × 10^−3^	1.08 × 10^−1^	1.75 × 10^−1^	2.52 × 10^−4^	2.36 × 10^−5^	8.74 × 10^−5^	1.29 × 10^−6^	3.64 × 10^−4^
	*M. excoriata*	4.79 × 10^−2^	2.15 × 10^−2^	3.96 × 10^−3^	5.55 × 10^−3^	9.15 × 10^−2^	1.70 × 10^−1^	2.90 × 10^−4^	3.23 × 10^−5^	9.81 × 10^−5^	1.09 × 10^−6^	4.22 × 10^−4^
	*M. procera*	4.73 × 10^−2^	1.06 × 10^−2^	4.61 × 10^−3^	7.43 × 10^−3^	1.13 × 10^−1^	1.83 × 10^−1^	2.87 × 10^−4^	1.59 × 10^−5^	1.31 × 10^−4^	1.34 × 10^−6^	4.35 × 10^−4^
		Cd	Cr	Cu	Ni	Pb	Total	Cd	Cr	Ni	Pb	Total
	Site 1	5.27 × 10^−2^	1.71 × 10^−2^	7.11 × 10^−3^	7.78 × 10^−3^	1.20 × 10^−1^	2.04 × 10^−1^	3.19 × 10^−4^	2.56 × 10^−5^	1.38 × 10^−4^	1.42 × 10^−6^	4.84 × 10^−4^
	Site 2	6.08 × 10^−2^	1.64 × 10^−2^	4.41 × 10^−3^	6.40 × 10^−3^	9.48 × 10^−2^	1.83 × 10^−1^	3.68 × 10^−4^	2.45 × 10^−5^	1.13 × 10^−4^	1.13 × 10^−6^	5.07 × 10^−4^
	Site 3	6.91 × 10^−2^	1.13 × 10^−2^	6.93 × 10^−3^	4.73 × 10^−3^	9.30 × 10^−2^	1.85 × 10^−1^	4.19 × 10^−4^	1.69 × 10^−5^	8.42 × 10^−5^	1.10 × 10^−6^	5.21 × 10^−4^
	Site 4	5.76 × 10^−2^	1.84 × 10^−2^	4.16 × 10^−3^	5.34 × 10^−3^	9.48 × 10^−2^	1.80 × 10^−1^	3.49 × 10^−4^	2.76 × 10^−5^	9.45 × 10^−5^	1.13 × 10^−6^	4.72 × 10^−4^
	Site 5	5.75 × 10^−2^	1.28 × 10^−2^	4.44 × 10^−3^	6.93 × 10^−3^	1.01 × 10^−1^	1.82 × 10^−1^	3.49 × 10^−4^	1.92 × 10^−5^	1.22 × 10^−4^	1.19 × 10^−6^	4.91 × 10^−4^
	Site 6	7.07 × 10^−2^	1.61 × 10^−2^	4.65 × 10^−3^	5.17 × 10^−3^	8.49 × 10^−2^	1.82 × 10^−1^	4.29 × 10^−4^	2.42 × 10^−5^	9.15 × 10^−5^	1.01 × 10^−6^	5.45 × 10^−4^

**Table 10:** Mean values of elements (i.e., Cd, Cr, Cu, Ni, and Pb) determined in mushroom samples and subsamples in terms of both species and collecting sites (expressed in mg·kg^−1^ d.w.).

		Cd	Cr	Cu	Ni	Pb
Collecting sites						
Site 1		1.108	0.985	3.062	1.147	1.845
Site 2		0.679	0.870	3.226	1.264	1.406
Site 3		0.326	0.795	2.965	1.448	0.667
Site 4		0.522	0.721	2.748	1.511	1.255
Site 5		0.785	0.755	3.155	1.055	1.310
Site 6		0.783	1.428	3.037	1.211	1.914
Mushroom species						
*Agaricus bisporus*		0.422	0.859	2.745	1.361	0.796
*Agaricus campestris*		0.905	0.450	2.830	0.954	1.636
*Armillaria mellea*		0.858	1.023	3.387	1.287	1.972
*Boletus edulis*		0.581	0.749	2.874	1.452	0.885
*Macrolepiota excoriata*		0.650	0.966	2.936	1.306	1.332
*Macrolepiota procera*		0.963	1.218	3.186	1.031	1.462
Subsamples						
*Agaricus bisporus*	Cap	0.620	0.774	3.457	1.072	0.576
	Stipe	0.223	0.943	2.033	1.649	1.016
*Agaricus campestris*	Cap	1.364	0.373	3.637	0.571	1.738
	Stipe	0.446	0.528	2.024	1.338	1.534
*Armillaria mellea*	Cap	1.158	1.220	4.132	1.268	2.134
	Stipe	0.558	0.827	2.642	1.305	1.809
*Boletus edulis*	Cap	0.924	0.845	3.453	1.062	0.682
	Stipe	0.238	0.653	2.294	1.843	1.088
*Macrolepiota excoriata*	Cap	1.096	0.906	3.608	0.918	1.274
	Stipe	0.205	1.025	2.265	1.694	1.391
*Macrolepiota procera*	Cap	1.605	1.616	3.513	1.016	1.783
	Stipe	0.321	0.819	2.860	1.047	1.140
Romanian Order no. 975/1998 (i.e., fresh vegetable)		0.100	nd*	5.000	nd*	0.500
Commission Regulation (EC) no. 2023/915 (i.e., Brassicaceae vegetables and wild mushrooms”)		0.200′ 0.500″	nd*	nd*	nd*	0.300′ 0.800″
Codex Alimentarius CODEX-STAN 193-1995 (i.e., Brassicaceae vegetables)		0.050	nd*	nd*	nd*	0.100

nd*—not specified value(s) in the regulations.

**Table 11:** Descriptive statistics of transfer factor (TF [%]) calculated for the mushroom subsamples (cap and stipe).

TF [%]		Cd	Cr	Cu	Ni	Pb
Cap	min	4.899	4.757	9.746	1.631	1.528
	max	76.774	115.648	33.984	26.534	35.200
	average	27.628	35.062	25.857	12.998	13.825
	median	24.952	23.802	26.958	12.167	11.506
	SD	18.359	29.532	6.417	6.313	9.773
Stipe	min	3.171	8.701	3.741	2.936	6.568
	max	19.529	69.880	30.505	35.221	27.396
	average	8.417	26.682	16.667	17.982	13.512
	median	6.428	21.270	17.204	19.150	13.344
	SD	5.290	16.891	6.026	8.675	5.169

**Table 12:** Mean values of estimated daily intake (*EDI*) and carcinogenic risk induced by lead (*CR_Pb_*) for each determined element (i.e., Cd, Cr, Cu, Ni, and Pb) in mushroom samples in terms of both species and collecting sites.

Adults	Estimated Daily Intake [mg·Day^−1^]					CR_Pb_
	Cd	Cr	Cu	Ni	Pb	
Collecting sites						
Site 1	0.111	0.099	0.306	0.115	0.184	1.57 × 10^−3^
Site 2	0.068	0.087	0.323	0.126	0.141	1.19 × 10^−3^
Site 3	0.033	0.080	0.296	0.145	0.067	5.67 × 10^−4^
Site 4	0.052	0.072	0.275	0.151	0.126	1.07 × 10^−3^
Site 5	0.078	0.075	0.315	0.105	0.131	1.11 × 10^−3^
Site 6	0.078	0.143	0.304	0.121	0.191	1.63 × 10^−3^
Mushroom species						
*Agaricus bisporus*	0.042	0.086	0.274	0.136	0.080	6.77 × 10^−4^
*Agaricus campestris*	0.090	0.045	0.283	0.095	0.164	1.39 × 10^−3^
*Armillaria mellea*	0.086	0.102	0.339	0.129	0.197	1.68 × 10^−3^
*Boletus edulis*	0.058	0.075	0.287	0.145	0.088	7.52 × 10^−4^
*Macrolepiota excoriata*	0.065	0.097	0.294	0.131	0.133	1.13 × 10^−3^
*Macrolepiota procera*	0.096	0.122	0.319	0.103	0.146	1.24 × 10^−3^
Children	Estimated daily intake [mg·day^−1^]					CR_Pb_
	Cd	Cr	Cu	Ni	Pb	
Collecting sites						
Site 1	0.033	0.030	0.092	0.034	0.055	4.70 × 10^−4^
Site 2	0.020	0.026	0.097	0.038	0.042	3.58 × 10^−4^
Site 3	0.010	0.024	0.089	0.043	0.020	1.70 × 10^−4^
Site 4	0.016	0.022	0.082	0.045	0.038	3.20 × 10^−4^
Site 5	0.024	0.023	0.095	0.032	0.039	3.34 × 10^−4^
Site 6	0.023	0.043	0.091	0.036	0.057	4.88 × 10^−4^
Mushroom species						
*Agaricus bisporus*	0.013	0.026	0.082	0.041	0.024	2.03 × 10^−4^
*Agaricus campestris*	0.027	0.014	0.085	0.029	0.049	4.17 × 10^−4^
*Armillaria mellea*	0.026	0.031	0.102	0.039	0.059	5.03 × 10^−4^
*Boletus edulis*	0.017	0.022	0.086	0.044	0.027	2.26 × 10^−4^
*Macrolepiota excoriata*	0.020	0.029	0.088	0.039	0.040	3.40 × 10^−4^
*Macrolepiota procera*	0.029	0.037	0.096	0.031	0.044	3.73 × 10^−4^
Tolerable intake level/ Recommended daily intake/ Acceptable risk level for CR_Pb_	0.025	0.025	0.900	1.000	0.250	1.00 × 10^−6^−1.00 × 10^−4^

For Cd and Pb are presented the values for tolerable intake level, calculated from the tolerable weekly intake (TWI) of 2.5 μg/kg b.m. and 25 μg/kg b.m., respectively, considering the average body mass = 70 kg; for Cr and Cu is presented the recommended daily intake; for Ni is presented the values for tolerable intake level.

**Table 13:** Mean values of daily intake metals (*DIM*) for each determined element (i.e., Cd, Cr, Cu, Ni, and Pb) in mushroom samples in terms of both species and collecting sites.

Adults	Daily Intake Metals [mg·kg^−1^·Day^−1^]				
	Cd	Cr	Cu	Ni	Pb
Collecting sites					
Site 1	0.0016	0.0014	0.0044	0.0016	0.0026
Site 2	0.0010	0.0012	0.0046	0.0018	0.0020
Site 3	0.0005	0.0011	0.0042	0.0021	0.0010
Site 4	0.0007	0.0010	0.0039	0.0022	0.0018
Site 5	0.0011	0.0011	0.0045	0.0015	0.0019
Site 6	0.0011	0.0020	0.0043	0.0017	0.0027
Mushroom species					
*Agaricus bisporus*	0.0006	0.0012	0.0039	0.0019	0.0011
*Agaricus campestris*	0.0013	0.0006	0.0040	0.0014	0.0023
*Armillaria mellea*	0.0012	0.0015	0.0048	0.0018	0.0028
*Boletus edulis*	0.0008	0.0011	0.0041	0.0021	0.0013
*Macrolepiota excoriata*	0.0009	0.0014	0.0042	0.0019	0.0019
*Macrolepiota procera*	0.0014	0.0017	0.0046	0.0015	0.0021
Childrens	Daily intake metals [mg·kg^−1^·day^−1^]				
	Cd	Cr	Cu	Ni	Pb
Collecting sites					
Site 1	0.0024	0.0021	0.0066	0.0025	0.0040
Site 2	0.0015	0.0019	0.0069	0.0027	0.0030
Site 3	0.0007	0.0017	0.0064	0.0031	0.0014
Site 4	0.0011	0.0015	0.0059	0.0032	0.0027
Site 5	0.0017	0.0016	0.0068	0.0023	0.0028
Site 6	0.0017	0.0031	0.0065	0.0026	0.0041
Mushroom species					
*Agaricus bisporus*	0.0009	0.0018	0.0059	0.0029	0.0017
*Agaricus campestris*	0.0019	0.0010	0.0061	0.0020	0.0035
*Armillaria mellea*	0.0018	0.0022	0.0073	0.0028	0.0042
*Boletus edulis*	0.0012	0.0016	0.0062	0.0031	0.0019
*Macrolepiota excoriata*	0.0014	0.0021	0.0063	0.0028	0.0029
*Macrolepiota procera*	0.0021	0.0026	0.0068	0.0022	0.0031

**Table 14:** Mean values of health risk index (*HRI*) and total target hazard quotient (*T_THQ_*) for each determined element (i.e., Cd, Cr, Cu, Ni, and Pb) in mushroom samples in terms of both species and collecting sites.

Adults	Health Risk Index					T_THQ_
	Cd	Cr	Cu	Ni	Pb	
Collecting sites						
Site 1	1.582	0.001	0.109	0.082	0.075	1.850
Site 2	0.971	0.001	0.115	0.090	0.057	1.235
Site 3	0.466	0.001	0.106	0.103	0.027	0.703
Site 4	0.746	0.001	0.098	0.108	0.051	1.004
Site 5	1.121	0.001	0.113	0.075	0.053	1.364
Site 6	1.119	0.002	0.108	0.086	0.078	1.394
Mushroom species						
*Agaricus bisporus*	0.602	0.001	0.098	0.097	0.032	0.831
*Agaricus campestris*	1.293	0.001	0.101	0.068	0.067	1.529
*Armillaria mellea*	1.225	0.001	0.121	0.092	0.080	1.520
*Boletus edulis*	0.830	0.001	0.103	0.104	0.036	1.074
*Macrolepiota excoriata*	0.929	0.001	0.105	0.093	0.054	1.183
*Macrolepiota procera*	1.375	0.002	0.114	0.074	0.060	1.624
Childrens	Health risk index					T_THQ_
	Cd	Cr	Cu	Ni	Pb	
Collecting sites						
Site 1	2.374	0.004	0.164	0.123	0.113	2.778
Site 2	1.456	0.004	0.173	0.135	0.086	1.854
Site 3	0.698	0.003	0.159	0.155	0.041	1.057
Site 4	1.118	0.003	0.147	0.162	0.077	1.507
Site 5	1.682	0.003	0.169	0.113	0.080	2.047
Site 6	1.678	0.006	0.163	0.130	0.117	2.093
Mushroom species						
*Agaricus bisporus*	0.904	0.004	0.147	0.146	0.049	1.249
*Agaricus campestris*	1.939	0.002	0.152	0.102	0.100	2.295
*Armillaria mellea*	1.838	0.004	0.181	0.138	0.121	2.282
*Boletus edulis*	1.245	0.003	0.154	0.156	0.054	1.612
*Macrolepiota excoriata*	1.394	0.004	0.157	0.140	0.082	1.777
*Macrolepiota procera*	2.063	0.005	0.171	0.111	0.089	2.439

**Table 15:** Descriptive statistics for soil samples.

Elements	Range	Minimum	Maximum	Mean	Standard Deviation	Variance
Cd	4.09200	2.32400	6.41600	4.3589474	1.08212258	1.171
Cr	4.41300	1.36000	5.77300	3.4620526	1.24062836	1.539
Cu	32.16600	11.80400	43.97000	16.0414737	8.30874606	69.035
Ni	8.36500	5.09900	13.46400	8.9914211	2.53208599	6.411
Pb	9.74700	8.35500	18.10200	10.4321579	2.32516232	5.406

**Table 16:** Descriptive statistics for mushroom samples.

Elements	Range	Minimum	Maximum	Mean	Standard Deviation	Variance
Cd	1.607	0.178	1.785	0.716	0.541	0.293
Cr	1.727	0.178	1.905	0.899	0.446	0.199
Cu	3.929	1.121	5.050	3.043	0.892	0.797
Ni	2.314	0.178	2.49200	1.268	0.528	0.279
Pb	3.027	0.178	3.20500	1.396	0.759	0.577

**Table 17:** Pearson correlation between analyzed HMs in all samples.

		Cd	Cr	Cu	Ni	Pb
Cd	Pearson Correlation	1	0.760 **	0.795 **	0.849 **	0.864 **
	Sig. (2-tailed)		0.000	0.000	0.000	0.000
Cr	Pearson Correlation		1	0.662 **	0.777 **	0.820 **
	Sig. (2-tailed)			0.000	0.000	0.000
Cu	Pearson Correlation			1	0.799 **	0.854 **
	Sig. (2-tailed)				0.000	0.000
Ni	Pearson Correlation				1	0.918 **
	Sig. (2-tailed)					0.000
Pb	Pearson Correlation					1
	Sig. (2-tailed)					

** Correlation is significant at the 0.01 level (2-tailed).

## Data Availability

The original contributions presented in this study are included in the article and Supplementary Materials. Further inquiries can be directed to the corresponding authors.

## Supplementary Materials

The following supporting information can be downloaded at: https://www.mdpi.com/article/10.3390/jof10120844/s1. This manuscript is sustained by additional useful information presented in the Supplementary Material S1—Soil samples and Supplementary Material S2—Mushroom samples.
